# Deep phenotyping of EHMT1 ankyrin repeat domain missense variants in Kleefstra syndrome by multi-tiered structural genomics analyses at atomic resolution

**DOI:** 10.1093/hmg/ddag094

**Published:** 2026-09-28

**Authors:** Karina L Bursch, Young-In Chi, Jacob Licklider, Davin R Jensen, Audrey E Catlin, Michael T Zimmermann, Gwen Lomberk, Raul A Urrutia, Brian C Smith

**Affiliations:** Linda T. and John A. Mellowes Center for Genomic Sciences and Precision Medicine, Medical College of Wisconsin, Milwaukee, WI, United States; Department of Biochemistry, Medical College of Wisconsin, Milwaukee, WI, United States; Linda T. and John A. Mellowes Center for Genomic Sciences and Precision Medicine, Medical College of Wisconsin, Milwaukee, WI, United States; Division of Research, Department of Surgery, Medical College of Wisconsin, Milwaukee, WI, United States; Linda T. and John A. Mellowes Center for Genomic Sciences and Precision Medicine, Medical College of Wisconsin, Milwaukee, WI, United States; Department of Biochemistry, Medical College of Wisconsin, Milwaukee, WI, United States; Linda T. and John A. Mellowes Center for Genomic Sciences and Precision Medicine, Medical College of Wisconsin, Milwaukee, WI, United States; Department of Biochemistry, Medical College of Wisconsin, Milwaukee, WI, United States; Linda T. and John A. Mellowes Center for Genomic Sciences and Precision Medicine, Medical College of Wisconsin, Milwaukee, WI, United States; Department of Biochemistry, Medical College of Wisconsin, Milwaukee, WI, United States; Linda T. and John A. Mellowes Center for Genomic Sciences and Precision Medicine, Medical College of Wisconsin, Milwaukee, WI, United States; Department of Biochemistry, Medical College of Wisconsin, Milwaukee, WI, United States; Data Science Institute, Medical College of Wisconsin, Milwaukee, WI, United States; Linda T. and John A. Mellowes Center for Genomic Sciences and Precision Medicine, Medical College of Wisconsin, Milwaukee, WI, United States; Division of Research, Department of Surgery, Medical College of Wisconsin, Milwaukee, WI, United States; Department of Pharmacology and Toxicology, Medical College of Wisconsin, Milwaukee, WI, United States; Linda T. and John A. Mellowes Center for Genomic Sciences and Precision Medicine, Medical College of Wisconsin, Milwaukee, WI, United States; Department of Biochemistry, Medical College of Wisconsin, Milwaukee, WI, United States; Division of Research, Department of Surgery, Medical College of Wisconsin, Milwaukee, WI, United States; Linda T. and John A. Mellowes Center for Genomic Sciences and Precision Medicine, Medical College of Wisconsin, Milwaukee, WI, United States; Department of Biochemistry, Medical College of Wisconsin, Milwaukee, WI, United States

**Keywords:** Epigenetics, Histone modification, Missense variant, Molecular genetics, Pathogenicity classification, Variant of uncertain significance

## Abstract

EHMT1 is a lysine methyltransferase that writes and reads histone methylation marks to repress transcription. Deleterious mutations in *EHMT1* cause Kleefstra syndrome (KLEFS1; OMIM #610253), a congenital neurodevelopmental disorder. Many of these affect the ankyrin repeat (ANKR) domain, which recognizes histone H3 lysine-9 methylation and mediates interactions with other epigenetic regulators. However, the functional consequences of EHMT1 missense variants within the ANKR domain remain poorly characterized, as nearly 50% are currently classified as variants of unknown significance (VUS). To address this gap, we performed a comprehensive structural genomics analysis to predict how 56 Kleefstra syndrome-associated missense variants alter EHMT1 ANKR domain structure and function. We integrated sequence-based pathogenicity algorithms with protein structural and dynamics metrics to generate variant molecular fitness scores. From these predictions, we selected 16 representative variants for experimental validation. Using differential scanning fluorimetry and circular dichroism, we quantified variant-specific effects on ANKR domain stability and secondary structure. We further assessed methyl-lysine binding using fluorescence polarization assays. By combining computational and biochemical analyses, we re-classified 80% of VUS as tolerated or damaging based on their effects on ANKR domain structural integrity and methyl-lysine binding. The resulting molecular fitness scores reveal mechanisms by which damaging variants disrupt EHMT1 ANKR domain stability, ligand binding, or both. Together, our integrated approach refines EHMT1 variant annotation and provides mechanistic insights that enhance current tools for variant interpretation in Kleefstra syndrome, which can be translated to missense variants in other epigenetic proteins and rare diseases.

## Graphical Abstract

**Graphical Abstract:**
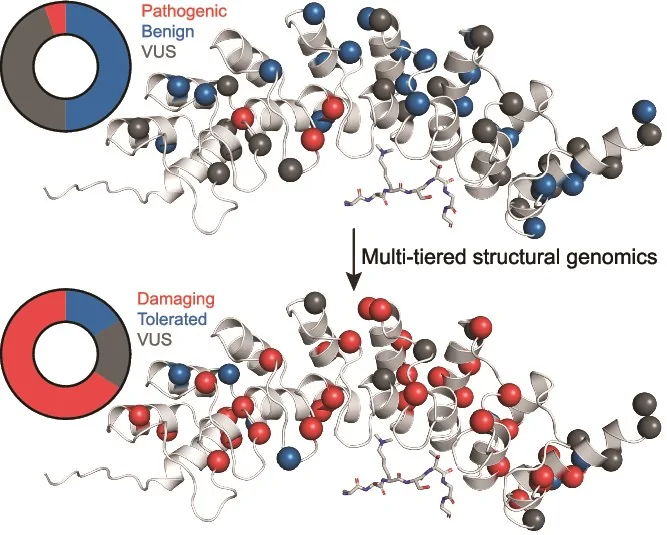

## Introduction

The euchromatic histone lysine methyltransferase 1 (*EHMT1*) gene encodes the G9a-like protein, EHMT1, a member of the Su(var)3-9, Enhancer-of-zeste and Trithorax (SET) domain-containing lysine methyltransferase family [1, 2]. EHMT1 contains three major functional domains: an *N*-terminal transactivation domain, an ankyrin repeat (ANKR) domain, and a *C*-terminal catalytic SET domain (Figure 1A) [1, 2]. The SET domain functions as an epigenetic writer [3] that catalyzes the mono- and di-methylation of histone H3 lysine-9 (H3K9me1 and H3K9me2) [2, 4, 5], whereas the ANKR domain acts as an epigenetic reader [3] of H3K9me1/me2 [2, 4–6]. In addition to its reader function, the ANKR domain facilitates assembly of higher-order epigenetic regulatory complexes to repress gene transcription [2, 4, 5]. Therefore, the ANKR domain is critical for maintaining the structure and function of the full-length EHMT1 protein.

**Figure 1:**
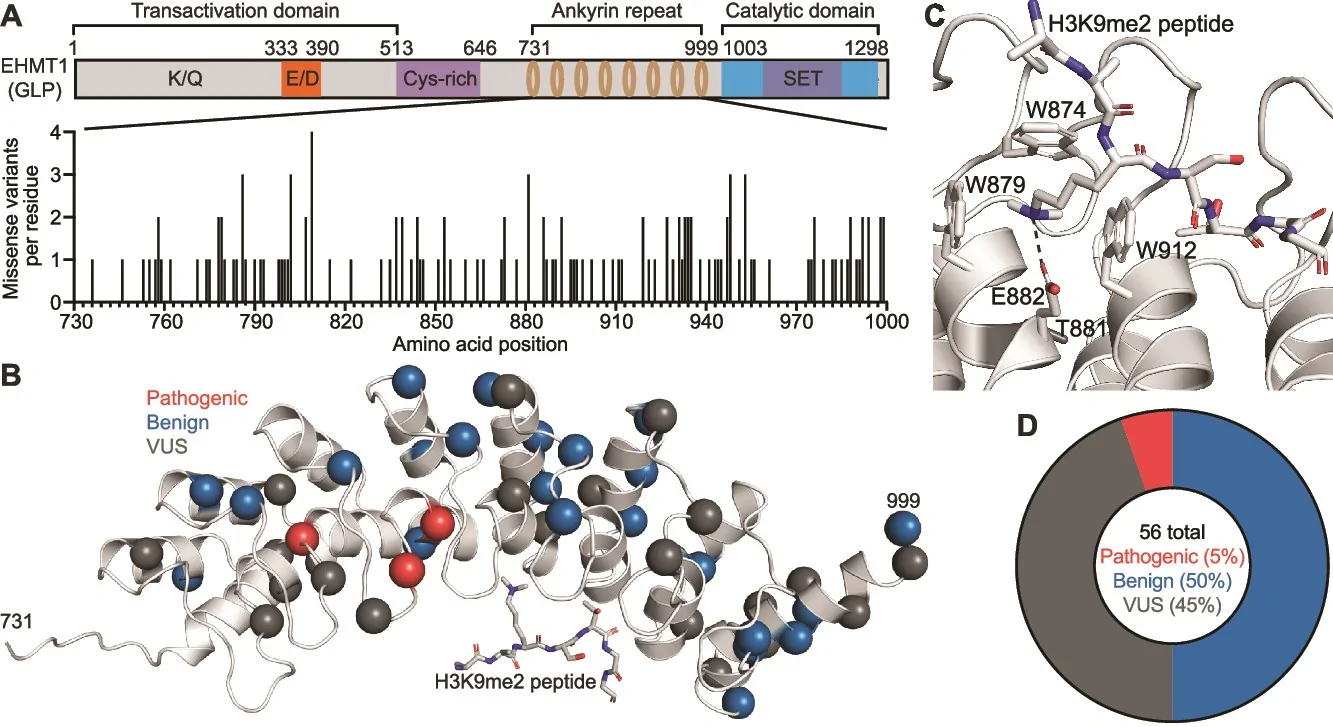
Kleefstra syndrome-associated missense variants are distributed across the EHMT1 ANKR domain. (A) EHMT1 domain structure and distribution of Kleefstra syndrome missense variants in the EHMT1 ANKR domain. The reported independent missense variants per residue were obtained from ClinVar [29]. (B) Amino acid residue positions of the 56 variants analyzed in this study, mapped onto the EHMT1 ANKR domain and colored by ClinVar annotation (blue: benign variants; gray: variants of uncertain significance (VUS); red: pathogenic variants). (C) The EHMT1 ANKR domain aromatic cage binds methylated histone lysine residues. Key residues involved in ligand coordination and nearby residue T881 (Kleefstra variants T881A/I) are shown as sticks. (D) Pie chart of the 56 Kleefstra syndrome missense variants analyzed in this study, grouped by ClinVar annotations.

ANKR domains belong to the alpha-solenoid family of protein-protein interaction modules characterized by repeating groups of anti-parallel α-helices [7–9]. Each ~33 residue repeat contains a conserved GXTPLHXA sequence, where X is any amino acid [10–12]. Several conserved residues in this sequence maintain the structural integrity of the ANKR domain by facilitating contacts within and between repeating units [10–12]. In contrast, variable residues at other positions mediate protein-protein interactions [10–12]. The EHMT1 ANKR domain comprises eight such tandem repeats joined by connecting loops (Figure 1B) [6, 13]. Together, these repeats bind H3K9me1 and H3K9me2 through a concave ligand-recognition surface formed by an aromatic cage of three Trp residues and a negatively-charged Glu residue (Figure 1B-C) [6]. These structural features enable specific methyl-lysine recognition and binding through a network of conserved aromatic and acidic residues.

As a lysine methyltransferase and an epigenetic reader, EHMT1 plays an essential role in epigenetic regulation during early embryonic development [4, 14, 15]. *De novo* or germline *EHMT1* mutations cause Kleefstra syndrome (KLEFS1; OMIM #610253) (KS), a neurodevelopmental disorder characterized by global developmental delay (HPO:0001263), intellectual disability (HPO:0001249), microcephaly (HPO:0000252), protruding tongue (HPO:0010808), and synophrys (HPO:0000664) (eyebrows meeting in the center of the face) [13, 16]. Although the effects of some pathogenic *EHMT1* mutations on SET domain structure and function have been investigated [1, 17], the contributions of ANKR domain missense mutations to KS pathogenesis remain largely unknown. Therefore, understanding how mutations disrupt ANKR domain structure and function is crucial for elucidating disease mechanisms.

To accelerate the medical genetic understanding of how missense variants contribute to KS and other rare diseases, many computational algorithms have been developed to predict variant effects using protein sequence features (2D) [18–22]. These methods typically classify variants as benign, pathogenic, or variant of uncertain significance (VUS) [18–22]. However, sequence-based predictions may be limited by insufficient structural information [19, 23] or an inability to assess functional alterations in biologically relevant mechanisms [21]. To address these limitations, we previously integrated multiple existing 2D prediction tools within a multi-tiered *in silico* structural genomics framework [1, 24–27] and demonstrated that domain-level mutational studies provide insights applicable to full-length proteins [28]. Here, we advance this framework to evaluate understudied EHMT1 ANKR domain missense variants using an integrated 2D-3D-4D approach that incorporates sequence analysis (2D), structural modeling (3D), and molecular dynamics-based metrics (4D). We experimentally interrogated predicted variant effects on ANKR domain stability and H3K9me1/me2 binding using differential scanning fluorimetry, circular dichroism, and fluorescence polarization. Integrating these quantitative biophysical data with *in silico* metrics allowed us to refine ClinVar annotations for 80% of the EHMT1 ANKR domain missense variants previously labeled as VUS. The results of our study demonstrate that integrated structural genomics analyses provide mechanistic resolution for EHMT1 variant interpretation, generating actionable hypotheses about their contributions to KS and enabling future studies to connect variant-induced structural perturbations to downstream transcriptional abnormalities underlying disease pathogenesis.

## Results

### Analysis of 119 Kleefstra syndrome variants reveals a broad distribution across the EHMT1 ankyrin repeat domain

To assess the mutational landscape of KS within the EHMT1 ANKR domain, we extracted all 119 domain-specific missense variants currently annotated in ClinVar [29] (Figure 1A). From this set, we selected 56 variants for detailed computational analysis, ensuring representation across the full length of the ANKR domain and across all three primary ClinVar annotation categories (benign, VUS, and pathogenic) (Figure 1B, 1D). Notably, missense variants do not cluster within discrete regions of the domain, indicating an absence of ANKR hot spots (Figure 1B). Only two variants (T881A/I) are located near the aromatic cage (W874, W879, and W912) that mediates recognition of methyl-lysine residues on target substrates (Figure 1C) [6]. Clinical variant interpretation guidelines recommend including functional impact predictions when annotating protein-coding variants [18, 22]. However, 25 of the 56 variants we analyzed (45%) are currently classified as VUS, reflecting limited clinical evidence and incomplete understanding of their molecular contributions to disease (Figure 1D). Thus, we sought to enhance the annotation of these 56 KS-associated missense variants by defining their predicted impact on the structure and function of the EHMT1 ANKR domain.

### A multi-tiered analysis integrating sequence, structure, and dynamic predictions identifies complementary metrics for variant impact assessment

To evaluate the effects of the 56 missense variants on EHMT1 ANKR domain stability and function, we first applied sequence-based (2D) missense variant impact prediction tools [18–22], including PolyPhen2 [30], Rhapsody [31], Protein Variation Effect Analyzer (PROVEAN) [32], and Rare Exome Variant Ensemble Learner (REVEL) [33] (Figure 2A, Table S1). Several of these 2D algorithms are recommended for clinical use by professional societies such as the American College of Medical Genetics and Genomics, the Association for Molecular Pathology, and the American Society of Clinical Oncology [18, 22]. We next assessed the same variants using structure-based (3D) metrics, calculating predicted changes in protein folding free energy and local structural perturbations as proxies for protein stability [34] (Figure 2A, Table S1). We also included energetic frustration as a 3D metric, which probes the energetic favorability of intermolecular contacts and can indicate biologically important regions, such as binding or allosteric sites (Figure 2A, Table S1) [35]. Collectively, this integrative approach provides complementary insights into how missense variants affect EHMT1 ANKR domain stability and functional integrity, advancing our understanding of their potential molecular contributions to disease.

**Figure 2:**
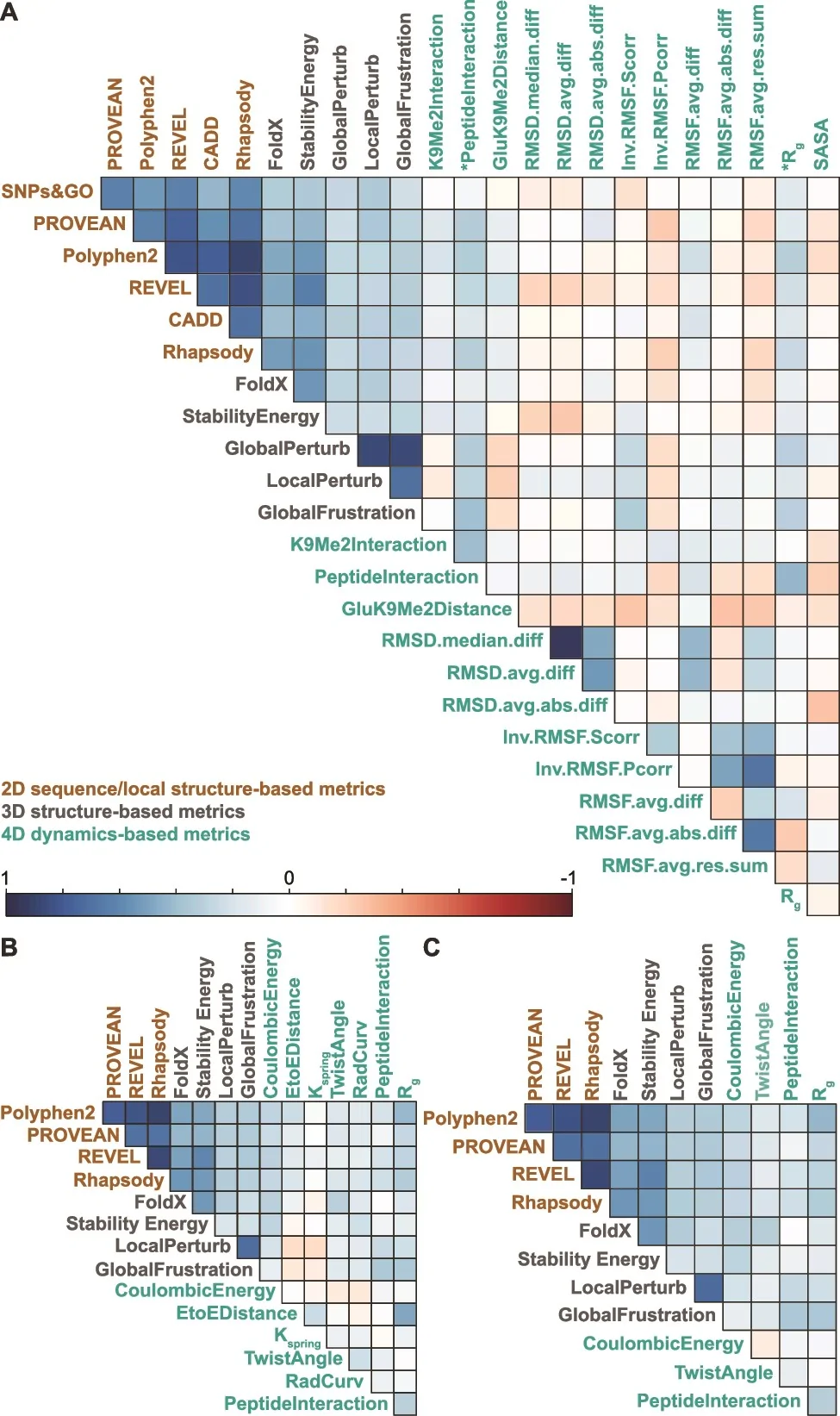
Integrated 2D, 3D, and 4D structural genomics analyses identify complementary metrics for evaluating the impacts of missense variants in Kleefstra syndrome. (A) Correlation matrix of individual metrics from the 2D, 3D, and 4D layers of the integrated structural genomics analysis. Asterisks indicate the two 4D metrics that exhibit the strongest correlation with metrics from the 2D and 3D analysis layers. (B) Correlation matrix of the 8 selected individual 2D and 3D metrics with 7 4D metrics. (C) Cross-correlation matrix of the 12 final metrics (4 per analysis layer) selected for molecular fitness score calculations. In all panels, 2D metrics are colored brown, 3D metrics are colored gray, and 4D metrics are colored green.

KS-associated EHMT1 ANKR domain missense variants were also examined using several molecular dynamics-based (4D) analyses. This included root mean square deviation (RMSD) and root mean square fluctuation (RMSF), which report on the global and residue-level dynamic stability of the protein, respectively [36] (Figure 2A, Table S1). Because the ANKR domain adopts a dynamically twisting solenoid structure [37–39], we additionally calculated the radius of gyration (R_g_) [40] and solvent-accessible surface area (SASA) [41], along with time-resolved ligand interaction energies (Figure 2A, Table S1). Following our established approach [1, 24–27], we used these combined 3D and 4D assessments to quantify the effects of KS-associated missense variants on ANKR domain stability and function, informed by either crystal structures deposited in the Protein Data Bank (PDB) (stressed) or energy-minimized models (relaxed).

We next assessed the congruency among all individual scores to identify the most informative metrics from each level (sequence/structure/dynamics; 2D/3D/4D) of our integrated analysis (Figure 2A-C). Interestingly, many scores showed varying degrees of correlation within and across these analytical tiers (Figure 2A). For instance, 3D scores generally aligned well with 2D scores (Figure 2A). In contrast to our previous findings with the catalytic EHMT1 SET domain, where RMSD and RMSF-related 4D scores exhibited modest congruence (*r* ≤ 0.32) with 2D and 3D scores [1], only peptide interaction energies and R_g_ among the 4D dynamics-based scores correlated with most 2D and 3D metrics (Figure 2A). The correlation of R_g_ with these metrics indicates the importance of maintaining the characteristic ANKR domain structure [10–12] for preserving overall protein function (Figure 1B). Therefore, these results demonstrate that 2D and 3D metrics provide substantial predictive value for assessing the potential structural and functional impacts of missense variants within the EHMT1 ANKR domain, while select 4D features offer complementary mechanistic insight.

To broaden our 4D analytical framework for the 56 missense variants, we measured Coulombic energy values as proxies for surface electrostatic and potential energies [42], features important for protein-protein interactions [43] characteristic of ANKR domains [10–12] (Figure 2B). We also evaluated motions associated with alpha-solenoid proteins [37–39] by calculating geometry-based parameters, including ANKR domain twist angles, radii of curvature, end-to-end distances for nano-spring compression, and elasticity spring constants (K_spring_), all derived from molecular dynamics trajectories (Figure 2B) [37–39, 44]. These geometry- and electrostatics-based scores showed modest correlations (*r* = 0.10 – 0.42) with other 2D and 3D impact metrics, except for K_spring_. To maintain balanced representation across analytical layers, only twist angles, Coulombic energies, histone peptide interactions, and R_g_ were included as 4D metrics in the overall impact scoring (Figure 2C). Together, these results demonstrate that select 3D and 4D features provide informative *in silico* readouts of established ANKR structural and functional characteristics. Therefore, integrating these 3D and 4D metrics with 2D prediction algorithms enhances the mechanistic interpretation of previously unresolved VUS and improves predictions of how missense variants alter EHMT1 ANKR domain stability and ligand binding.

### An integrated molecular fitness scoring system improves Kleefstra syndrome-associated EHMT1 ankyrin repeat domain missense variant classification accuracy

To minimize bias from any single analytical layer in evaluating the impacts of KS-associated missense variants, we selected four metrics from each layer (2D, 3D, and 4D) that showed concordance across the whole analysis framework (Figure 2B, Table S1). To standardize the units and ranges of these metrics and improve the translational utility of our integrated structural genomics approach, the raw outputs were first converted to Z-scores and then to a 0-1 scale [25], similar to the output formatting used by many 2D prediction algorithms [18–22]. Final molecular fitness scores for each EHMT1 ANKR domain missense variant were generated by combining the 12 normalized metrics with equal weights, followed by averaging (Table S1). Next, we compared the 2D sequence-based scores with the combined 3D/4D structure- and dynamics-derived scores and found a moderate correlation (*r* = 0.62) (Figure S1, Table S1). These results indicate that each scoring layer contributes complementary information to the overall evaluation of the ANKR domain missense variants. In summary, 2D scores provide quick estimates of variant molecular fitness. In contrast, the integrated 3D/4D metrics offer mechanistic insights into whether a variant is likely to be functionally tolerated or damaging to ANKR domain structure and function.

### Biophysical characterization reveals diverse effects of Kleefstra syndrome-associated variants on structural integrity and methyl-lysine ligand binding

To evaluate the molecular fitness predictions generated for the 56 missense variants, we recombinantly expressed and purified 16 representative variants for biophysical analyses. These variants were selected to include a minimum of three variants from each ClinVar annotation group and to provide coverage across the entire ANKR domain (Figure 3A). Variants T881A/I were also selected because of their proximity to the aromatic cage (Figure 1C), where substitutions may disrupt ligand binding functions. Additional variants, A780T, H844Y, G896D, and L976R, were chosen because they perturb residues in the conserved ANKR domain sequence motif (GXTPLHXA) [10–12]. Consistent with the structural importance of H844 [10–12, 17], the H844Y variant could not be purified due to low protein solubility. Additionally, we designed P709L as an *N*-terminal structural control, as variants in this region were expected to have minimal effects on domain stability or structural integrity. We also included a non-KS-associated cancer variant K733R [45]. Finally, W879A and E882A were included as functional controls because they directly disrupt aromatic cage-mediated methyl-lysine ligand binding (Figure 3B).

**Figure 3:**
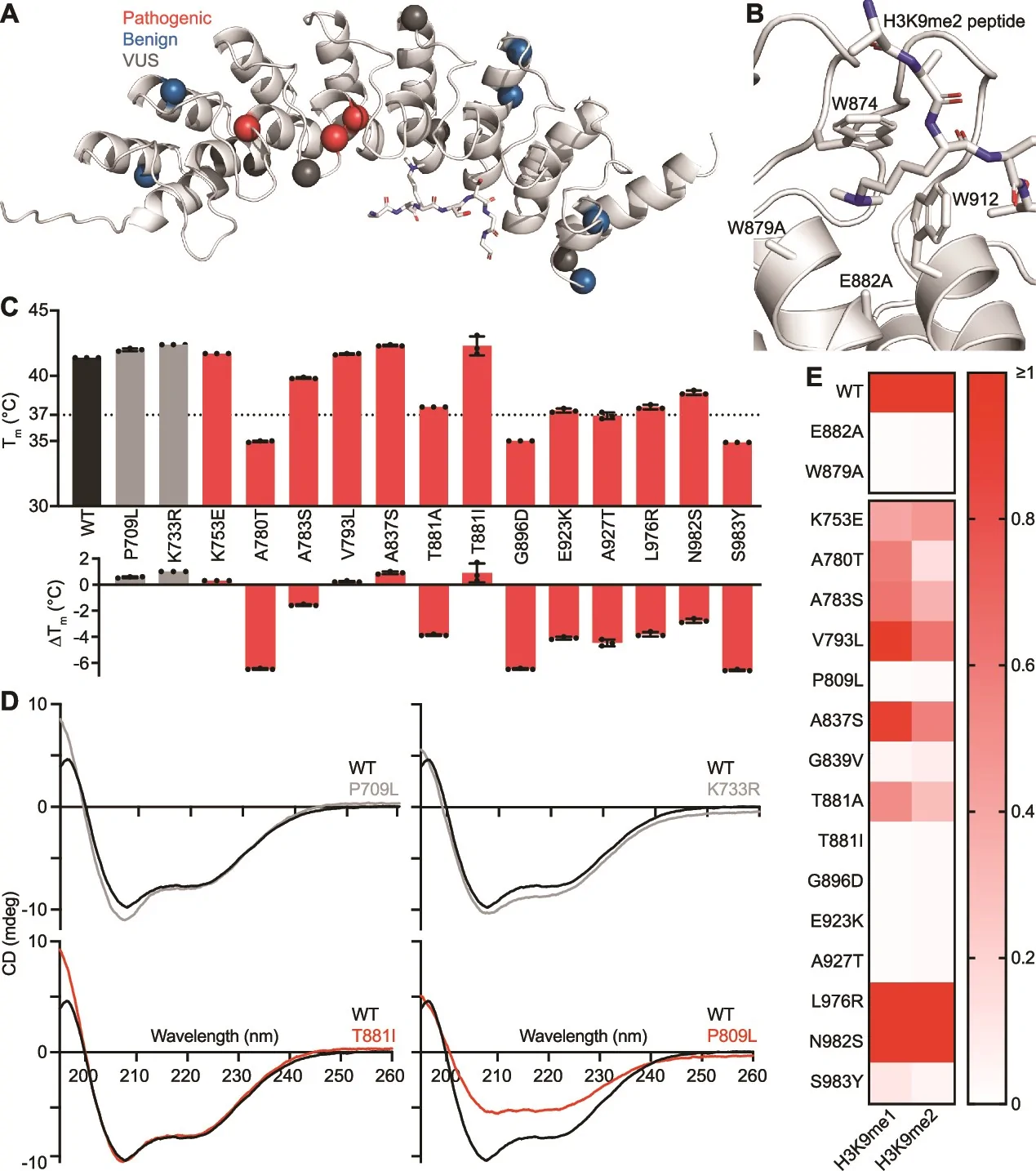
Representative Kleefstra syndrome-associated missense variants exhibit variable impacts on EHMT1 ANKR domain structure and function *in vitro*. (A) Amino acid residue positions of the 16 Kleefstra syndrome missense variants selected for biophysical analysis, colored by ClinVar annotation (blue: benign; gray: VUS; red: pathogenic). (B) Functional control variants W879A and E882A in the EHMT1 ANKR domain aromatic cage, shown as sticks. (C) Wild-type and missense variant EHMT1 ANKR domain *T*_m_ determined by nanoDSF (wild-type shown in black, and structural control variants shown in gray, Kleefstra syndrome-associated missense variants shown in red); *∆T*_m_ of variants compared to the wild-type is also shown. Error bars represent SD; n = 3 for all samples. (D) CD spectra of structural control variants P709L and K733R (gray) and Kleefstra syndrome-associated missense variants T881I and P809L (red) compared to the wild-type EHMT1 ANKR domain (black). (E) Heat map indicates FC of variant binding (*K*_d_) to H3K9me1 or H3K9me2 peptides (100 nM) relative to the wild-type (0.04 – 100 μM) identified from FP assays.

We first employed nano-differential scanning fluorimetry (nanoDSF) to determine melting temperatures (T_m_), a widely used proxy for protein stability [46, 47]. Most KS-associated missense variants exhibited thermal destabilization relative to the wild-type ANKR domain (∆T_m_ up to approximately -6 °C) (Figure 3C, Table S2). Two variants, P809L and G839V, did not yield measurable T_m_ values, consistent with substantial structural destabilization (Table S2). In contrast, four missense variants (K753E, V793L, A837S, and T881I) and both structural controls (P709L and K733R) had T_m_ values comparable to wild-type (Figure 3C, Table S2). Notably, eight variants had T_m_ values below (A780T, G896D, S983Y) or within 2 °C (T881A, E923K, A927T, L976R, N982S) of physiological temperature (37 °C) (Figure 3C, Table S2). Therefore, several KS-associated missense variants are likely to be fully or partially unfolded under physiological conditions.

Subsequently, we evaluated secondary structure using circular dichroism (CD), which reports α-helical content via characteristic minima at 208 and 222 nm [48, 49]. The CD spectra demonstrated that the canonical α-helical architecture of the ANKR domain remained intact for nearly all variants and both structural controls (Figures 3D and S2). As expected from the high structural destabilization in nanoDSF assays (Table S2), P809L and G839V exhibited reduced α-helical content indicative of disrupted secondary structure integrity (Figures 3D and S2). These findings are consistent with both our prior work and other studies on P809L [17, 50]. Taken together, the broad retention of secondary structure indicates that most variants induce local rather than global structural perturbations [28, 34].

Because the ANKR domain functions as an epigenetic reader [3] of H3K9me1/me2 and contributes to transcriptional regulation [2, 4–6], we assessed variant effects on binding to 5-tetramethylrhodamine (TAMRA)-labeled H3K9me1 and H3K9me2 histone tail peptides using fluorescence polarization (FP) [51]. Most variants, including both functional controls (W879A and E882A), demonstrated minimal binding to both peptides (Figures 3E and S3, Table S3). Seven variants retained robust binding (Figures 3E and S3, Table S3). Consistent with prior studies on the wild-type EHMT1 ANKR domain [6], all variants with preserved methylated histone peptide binding exhibited a tighter binding affinity for H3K9me1 than H3K9me2 (Table S3). These results indicate that a substantial subset of the tested missense variants disrupt ligand recognition by the ANKR domain, shedding light on how variant-induced structural alteration may impair EHMT1 reader function in the context of KS.

### Integrated scoring and mechanistic profiling refine interpretation of KS-associated EHMT1 ANKR domain variants

Since all EHMT1 ANKR domain missense variants analyzed in this study, excluding the designed structural and functional controls P709L, K733R, W879A, and E882A, occur in individuals with confirmed KS, their existing ClinVar annotations may not accurately reflect their functional consequences [52]. To provide a domain-specific, mechanism-informed classification scheme, we redefined the variant impact categories as tolerated, VUS, or damaging to directly describe the predicted and experimentally supported effects on ANKR domain stability and methyl-lysine binding based on their integrated molecular fitness score (IMFS) generated from the integrated structural genomics analysis (Table S1). To optimize the use of IMFS to annotate EHMT1 ANKR domain missense variants as tolerated, VUS, or damaging relative to structural integrity and histone peptide binding, we employed a receiver operating characteristic (ROC) curve analysis [53] (Figure S4, Table S4) based on H3K9me1 peptide binding (variant binding with H3K9me1 FC *K*_d_ ≥ 0.5). Since biophysical H3K9me1 binding was used as ROC input to define the IMFS thresholds for variant classification, the inclusion of a computational peptide interaction energy term in the IMFS is partly self-referential. Nonetheless, the ROC curve analysis yielded an area under the curve of 0.86 (Figure S4, Table S4), demonstrating that IMFS informed by H3K9me1 peptide binding have reliable predictive power. Based on this analysis, EHMT1 missense variants were assigned to three annotation categories for variant impact prediction (IMFS<0.2 tolerated; 0.2<IMFS<0.28 VUS; IMFS>0.28 damaging) (Figure S4, Table S4). We then plotted molecular fitness scores for all 56 variants (Table S1) against their original ClinVar annotations (Figure 4A).

**Figure 4:**
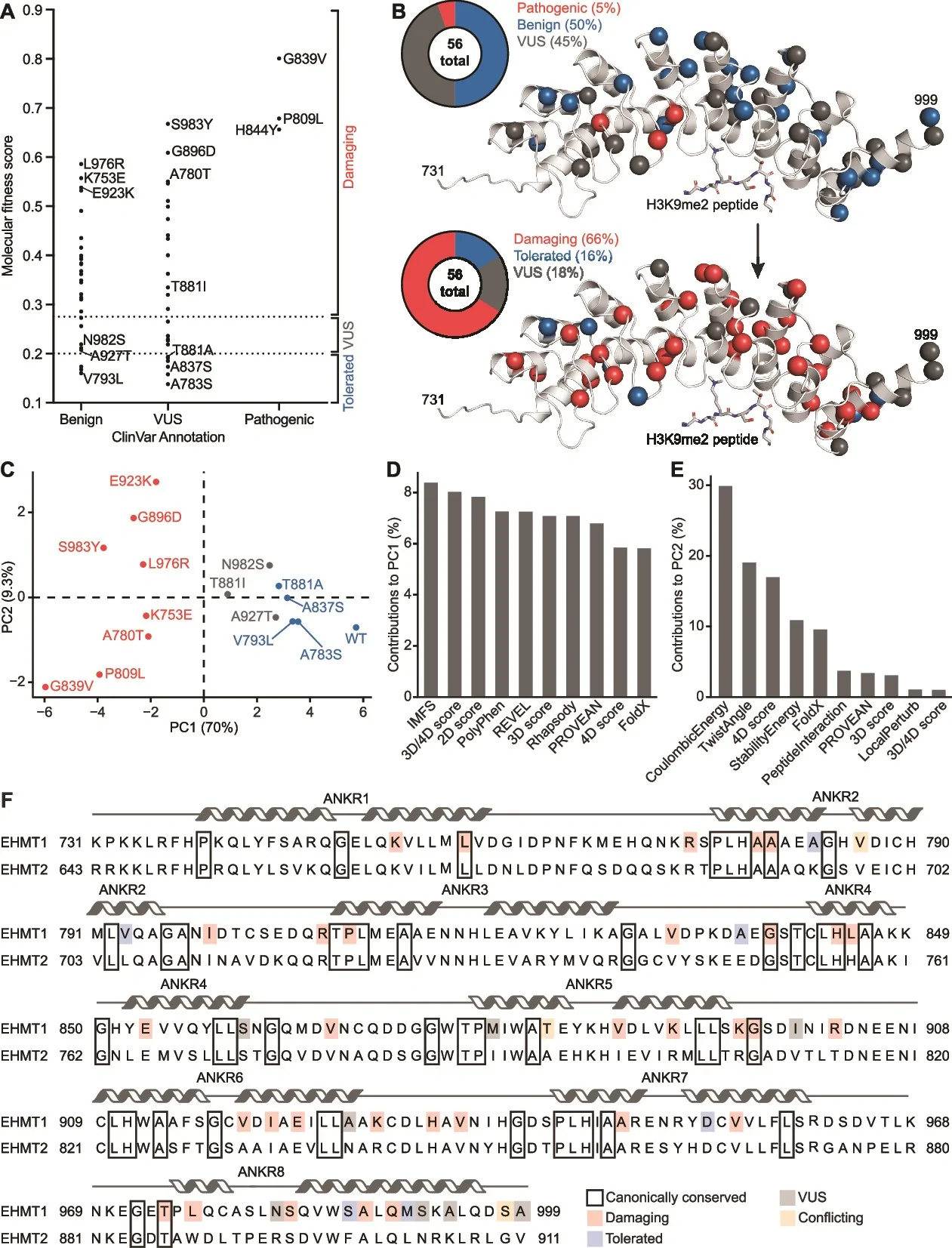
Integrated molecular fitness scoring refines the classification of Kleefstra syndrome-associated missense variants and identifies structural features underlying variant impact. (A) Graph of Kleefstra syndrome-associated missense variant molecular fitness scores relative to initial ClinVar classifications. Black dotted lines indicate the cutoffs set for the revised impact annotation categories (IMFS<0.2 tolerated; 0.2<IMFS<0.28 VUS; IMFS>0.28 damaging). Labeled variants indicate those selected for biophysical studies. (B) Pie charts of the 56 Kleefstra syndrome missense variants analyzed in this study, grouped by ClinVar annotations (blue: benign; gray: VUS; red: pathogenic) and by revised impact annotations (blue: tolerated; gray: VUS; red: damaging). Amino acid residue positions of the variants analyzed in this study are mapped onto the EHMT1 ANKR domain and colored by ClinVar annotations and revised impact annotations as above. (C) PCA of EHMT1 ANKR domain variants with computational and biophysical data from the integrated structural genomics analysis as inputs for variant clustering. (D) Contribution of the top 10 metrics to the first principal component of the PCA plot. (E) Contribution of the top 10 metrics to the second principal component of the PCA plot. (F) *S*tructure-based sequence alignment of the EHMT1 and EHMT2 ANKR domains, with the position of the eight tandem repeating units of antiparallel α-helices (gray) shown above. Positions of amino acid residues analyzed in this study are colored by missense variant revised impact annotations. Tan highlighting indicates residues where missense variants affecting the same amino acid residue have different impact annotations. Black boxes indicate canonically conserved ankyrin repeat residues [12].

Using these refined categories, 16% of the 56 KS-associated variants were classified as tolerated with respect to ANKR domain structure and function (Figure 4A-B). The Genome Aggregation Database (gnomAD) is a collection of sequencing data primarily obtained from individuals included as healthy controls in disease-focused studies [54]. The gnomAD database is widely used to estimate and compare allele frequencies in the context of rare disease diagnosis efforts [54, 55]. KS-associated variant I899V is present in gnomAD (v. 4.1.0) with a higher allele frequency (>4 × 10^-4^) and was also predicted to be a VUS in our analysis (Table S1). Therefore, I899V may contribute less to KS pathogenesis, as it is present in presumed healthy individuals in the general population. The revised annotations further indicated that 66% of the variants were damaging (Figure 4A-B), including the three variants (P809L, G839V, H844Y) previously annotated as pathogenic in ClinVar. In total, 18% of the 56 variants remained classified as VUS, representing a 2.5-fold improvement over the 45% annotated initially as VUS in ClinVar (Figure 4A-B). Specifically, 25 variants were previously annotated as VUS in ClinVar; using IMFS, 6 were re-annotated as tolerated and 14 as damaging (Table S1). Overall, 10 of the 56 total KS variants are considered VUS after re-annotation; 5 of these variants were previously annotated as VUS, and 5 previously benign variants were re-annotated as VUS (Table S1). Notably, our scoring system also corrected cases where the biophysical data conflicted with ClinVar annotations. For example, E923K and L976R, annotated as benign and likely benign, respectively (Table S1), were reclassified as damaging based on their IMFS (Table S1). Both variants were destabilized by ~4 °C relative to the wild-type ANKR domain (Figure 3C, Table S2) and entirely lost H3K9me1/me2 binding (Figure 3E, Figure S3, Table S3), consistent with their revised classifications.

We next evaluated how the integrated structural genomics analysis identifies variants with similar computational characteristics using principal component analysis (PCA). PCA demonstrated that variants annotated as damaging, VUS, or tolerated cluster into unique groups based on specific computational profiles (Figure 4C, Table S5). Notably, IMFS provided the greatest contributions to the first principal component, followed by 3D/4D and 2D scores (Figure 4D, Table S5), indicating that IMFS have high utility for distinguishing tolerated variants from damaging variants in the context of mechanistic variant interpretation. Coulombic energy (a 4D metric), twist angle (a 4D metric), and 4D scores provided the greatest contributions to the second principal component (Figure 4E, Table S5). We also assessed the correlation between scores from different levels of the integrated structural genomics analysis with select biophysical metrics on ANKR domain stability and ligand binding (Figure S5). For thermal stability (∆T_m_), 2D scores showed moderate correlation (*r^2^* = 0.33), while 4D and IMFS showed higher correlations (*r^2^* = 0.36-0.37) (Figure S5). Similarly, for ligand binding, quantified as FC in binding (*K*_d_) relative to wild-type, 2D scores correlated (*r^2^* = 0.37-0.40), whereas 4D and integrated scores again demonstrated stronger correlations (*r^2^* = 0.46-0.51) (Figure S5). Taken together, these results indicate that integrating sequence-based (2D), structure-based (3D), and dynamics-based (4D) metrics with experimental biophysical measurements improves the mechanistic resolution of ANKR domain variant assessment.

Following our previous work [25], we used differences between 3D and 4D scores to infer mechanisms of disruption for the variants classified as damaging (Table S1). Variants in which 3D scores exceeded 4D scores by >0.2 were categorized as structural variants (SVs), indicating a predominant disruption of domain stability and structural integrity. Conversely, those with 4D scores more than 0.2 higher than 3D scores were designated dynamic variants (DVs), suggesting impaired ligand-binding dynamics as the primary defect. Variants with score differences below 0.2 were considered structural/dynamic variants (SDVs), consistent with combined perturbation to both domain stability and ligand binding. Applying these criteria to the ten variants annotated as damaging that were tested biophysically, G839V was classified as an SV, G896D and E923K as DVs, and the remaining variants (K753E, A780T, P809L, H844Y, T881I, L976R, and S983Y) as SDVs. These assignments aligned mainly with their biophysical behavior, with SVs and SVDs widely exhibiting global and/or local destabilization relative to the wild-type ANKR domain (Figures 3C-D and S2, Table S2) and DVs and SDVs broadly displaying reduced H3K9me1/2 binding (Figures 3E and S3, Table S3). Consistent with its SDV classification, H844Y also displayed low solubility and could not be purified. Therefore, the classification of H844Y as a damaging variant and an SDV is limited by reliance on computational metrics alone, as biophysical metrics could not be integrated into the IMFS for this variant. Taken together, these mechanistic assignments demonstrate how integrating 3D and 4D computational metrics with biophysical data refines the interpretation of KS-associated variants and enables more accurate, functionally informed annotations.

## Discussion

The EHMT1 ANKR domain functions as an epigenetic reader of H3K9me1/me2 and facilitates the formation of repressive chromatin complexes [1–6]. In line with these critical regulatory functions, germline or *de novo* mutations in *EHMT1* result in the neurodevelopmental disorder Kleefstra syndrome [13, 16]. Accurate evaluation of how missense variants affect protein structural integrity and function is, therefore, crucial for understanding their contributions to disease. Although widely used sequence-based 2D algorithms incorporate population frequency, sequence conservation, and computational predictions, they rely on limited structural information [18–22]. However, protein function is fundamentally governed by its tertiary structure, conformational dynamics, and molecular interactions [56, 57]. Reflecting this, a large portion of pathogenic variants disrupt protein stability (~65%) [58–60] or intermolecular interactions (~40%) [61–63]. Therefore, these considerations highlight the need to incorporate structural and dynamic information to achieve the mechanistic resolution needed for variant interpretation in a clinical setting.

The current study advances the molecular understanding of Kleefstra syndrome by applying a multi-tiered structural genomics analysis [1, 24–27] that integrates 2D prediction tools with 3D structural modeling and 4D molecular dynamics to evaluate the impact of 56 missense variants on the EHMT1 ANKR domain at atomic resolution. PCA and correlation analyses across analytical layers revealed structure- and dynamics-based features that complement existing sequence-level predictions (Figure 4, Figure S5). Integrating these computational metrics with experimental assessments of protein stability and methyl-lysine binding enabled us to refine variant classifications based on their effects on ANKR domain integrity and reader function. These insights cannot be captured solely by 2D approaches.

Developmental constraints provide additional context for the observed distribution of ANKR domain variation. Loss of full-length EHMT1 expression during development is embryonic lethal [14, 15]. Moreover, studies in mice show that substitutions in the aromatic cage residues corresponding to human W874, W879, and E882 are postnatally lethal [64]. These findings underscore the essential nature of the methyl-lysine binding interface and suggest that variants that severely disrupt ANKR domain reader function may be incompatible with human viability. In line with this, only a single aromatic cage variant (W912R) has been identified in individuals with Kleefstra syndrome [17] (Figure 1A). The W912R aromatic cage variant was first identified in a study that analyzed matched genomic and clinical phenotypic data from over 100 individuals with rare *EHMT1* variants [17]. The W912R variant exhibited decreased binding to methylated H3K9 peptides, yet retained thermal stability *in vitro* [17]. Accordingly, loss of EHMT1 ANKR reader function alone may be sufficient to cause Kleefstra syndrome [17]. Additionally, several patient-derived variants alter residues adjacent to the aromatic cage, such as T881A/I. Therefore, these variants likely weaken ANKR domain methyl-lysine binding but fall below the threshold required to cause embryonic or perinatal lethality.

Structural principles of ankyrin repeat domains further illuminate why specific variants exert destabilizing effects. The conserved GXTPLHXA motif and other core residues maintain the folding, curvature, and dynamics of the alpha-solenoid architecture [10–12]. In contrast, non-conserved residues support protein-specific interactions [10–12]. Consistent with ANKR structure-function relationships, nearly half of the variants that we purified and annotated as damaging in our study affected canonically conserved residues. In contrast, tolerated variants were confined to variable surface positions (Figure 4F). This alignment between evolutionary constraint and functional impact provides biological support for our classifications and illustrates how incorporating structural and dynamic features can help resolve uncertainty in ClinVar VUS calls.

By integrating these structural and dynamic metrics, we reclassified 80% of ClinVar VUS as either tolerated or damaging, demonstrating that domain-specific biophysical information improves the resolution of ambiguous variant annotations. In some cases, as with the missense variant A927T, 2D scores lowered the IMFS, masking damaging effects that became evident only when 3D and 4D metrics were incorporated (Table S1; Figure 2). These examples illustrate that, for certain tolerated variants, structure- and dynamics-based evaluations provide a more faithful depiction of variant impact than sequence-based predictors. At the same time, some variants classified as tolerated, such as T881A, exhibited isolated defects in stability or binding (Table S1; Figure 3), suggesting that even modest perturbations may still influence ANKR function in ways not captured by current scoring frameworks. Continued development of additional 3D and 4D metrics will strengthen the predictive capacity of this multi-tiered structural genomics approach and further reduce the likelihood of variant misclassification.

Based on computational and experimental analyses of EHMT1 ANKR domain stability and methyl-lysine binding, the damaging variants examined here may contribute to Kleefstra syndrome by impairing the ability of the ANKR domain to engage H3K9me1/2 and to support the chromatin interactions required for EHMT1 function. The ANKR domain participates in establishing and reinforcing H3K9-marked heterochromatin through its reader activity and through coordination with EHMT1-EHMT2 complexes, and disruptions to stability or ligand recognition are expected to weaken the formation and spreading of repressive chromatin domains that support proper transcriptional control [2–5]. In contrast, the variants we classify as tolerated with respect to ANKR domain stability and peptide binding may influence EHMT1 function through mechanisms not captured by the assays used here. Their presence in individuals with confirmed Kleefstra syndrome indicates that preserved domain-level biophysical properties do not exclude functional consequences *in vivo*. Indeed, nearly all EHMT1 missense variants in Kleefstra syndrome occur *de novo* and are inherited in an autosomal dominant manner, resulting in haploinsufficiency [13, 17]. Therefore, haploinsufficiency may explain the Kleefstra syndrome clinical phenotypes observed in patients with EHMT1 ANKR domain missense variants [17]. Accordingly, since our biophysical measurements rely on isolated histone peptides, tolerated variants may still have altered recognition of methylated lysines within intact nucleosomes, where DNA wrapping, histone-histone contacts, and chromatin compaction shape ANKR binding [65–67]. Tolerated variants that affect residues involved in protein-protein interactions [10–12] may disrupt EHMT1 assembly with other proteins critical for epigenetic regulation, such as EHMT2 [2, 4], origin recognition complex-associated protein (ORCA) [68], and nuclear factor kappa B (NF-κB) [69]. Tolerated variants may also affect the contributions of EHMT1 to the establishment and maintenance of heterochromatin in cellular contexts [6, 70, 71]. These considerations indicate that further mechanistic insight will require evaluating the remaining variants in the context of full-length EHMT1 and native chromatin, where ANKR-dependent binding integrates with SET-domain methyltransferase activity, protein–protein interactions, and higher-order chromatin architecture.

Taken together, our studies demonstrate that integrating structural and functional assessments of *EHMT1* missense variants with existing sequence-based predictive tools enhances the accuracy and mechanistic depth of variant interpretation in Kleefstra syndrome. By incorporating ANKR-specific functional metrics and 3D/4D biophysical measurements, this approach clarifies the molecular consequences of both damaging and tolerated variants. Such an integrated strategy will refine standardized variant interpretation protocols and guidelines for Kleefstra syndrome and other rare diseases, particularly those involving alpha-solenoid proteins and chromatin-modifying enzymes. More broadly, our results provide a foundation for interrogating clinically relevant variants in greater detail and establish a mechanistic basis for advancing medical genetics.

## Materials and methods

Detailed methods are available in the supplementary material.

## Supplementary Material

Supplementary_Data_ddag094

## Data Availability

MD simulation data were uploaded to the open-access MD data repository MDRepo [72] (accession numbers MDR00021551-MDR00021607). All other data and materials are available upon request.
