# Supplementary material for: Deep phenotyping of EHMT1 ankyrin repeat domain missense variants in Kleefstra syndrome by multi-tiered structural genomics analyses at atomic resolution
Source: Hum Mol Genet. 2026 Sep 28;35(20):ddag094. doi: 10.1093/hmg/ddag094 (PMC13626213; doi:10.1093/hmg/ddag094)
Supplement: Supplementary file 1 [file supplementary_data_ddag094.zip › Supplementary information_revised.pdf]

Supplementary Information

Deep phenotyping of EHMT1 ankyrin repeat domain missense variants in Kleefstra syndrome by multi-tiered structural genomics analyses at atomic resolution

Karina L. Bursch,[1,3#] Young-In Chi,[1,2#] Jacob Licklider,[1,3] Davin R. Jensen,[1,3] Audrey E. Catlin,[1,3] Michael T. Zimmermann,[1,2,4] Gwen Lomberk,[1,2,4] Raul A. Urrutia,[1,2,3]* and Brian C. Smith[1,3]*

[1]Linda T. and John A. Mellowes Center for Genomic Sciences and Precision Medicine, Medical College of Wisconsin, Milwaukee, WI
[2]Division of Research, Department of Surgery, Medical College of Wisconsin, Milwaukee, WI
[3]Department of Biochemistry, Medical College of Wisconsin, Milwaukee, WI
[4]Data Science Institute, Medical College of Wisconsin, Milwaukee, WI
[5]Department of Pharmacology and Toxicology, Medical College of Wisconsin, Milwaukee, WI

#Co-first authors
*Co-corresponding authors

Address correspondence to:
Raul A. Urrutia, MD, urrutia.raul@mayo.edu
Brian C. Smith, PhD, brismith@mcw.edu

Table of Contents

SUPPLEMENTARY MATERIALS AND METHODS **3**

SUPPLEMENTAL TEXT **10**

Text S1. R script used to evaluate solenoid geometry. 10
Text S2. R script used to generate the Figure 2 correlation matrices. 12
Text S3. R script used to generate the Figure 4 PCA plot. 14

SUPPLEMENTAL TABLES **16**

Table S1. Spreadsheet of the 12 selected individual prediction methods and group layer scores, scaled Z-scores, and molecular fitness scores. 16
Table S2. EHMT1 ANKR domain wild-type and missense variant T_m and ΔT_m values. 16
Table S3. Binding affinity (K_d) of EHMT1 ANKR domain wild-type and missense variants to H3K9me1 and H3K9me2 peptides. 17
Table S4. Sensitivity and specificity from ROC with IMFS and H3K9me1 peptide binding. 18
Table S5. Percent contributions of integrated structural genomics analysis metrics to the first two components of EHMT1 ANKR domain missense variant PCA. 19

SUPPLEMENTAL FIGURES **20**

Figure S1. Correlation between Kleefstra syndrome-associated missense variant 2D impact scores and 3D/4D impact scores. 20
Figure S2. Circular dichroism spectra of Kleefstra syndrome-associated EHMT1 ANKR domain missense variants. 21
Figure S3. FP titrations of the wild-type and missense variant EHMT1 ANKR domains (0.04 – 100 μM) binding to fluorescent 5-TAMRA-labeled H3K9me1 or H3K9me2 peptides (100 nM). 22
Figure S4. Receiver operating characteristic (ROC) analysis for score threshold determination. 23
Figure S5. Correlation between structural genomics analysis level scores and EHMT1 ANKR domain missense variant ligand stability and ligand binding as assessed by nanoDSF (ΔT_m) and FC (fold change of the K_d for H3K9me1 and H3K9me2 peptides compared to the wild-type EHMT1 ANKR domain, where K_d FC <2). 24
Figure S6. Representative RMSD graphs for replicate inclusion vs. exclusion. 25

SUPPLEMENTAL REFERENCES **26**

Supplementary Materials and Methods

Data collection and 2D missense variant impact prediction

Fifty-six KS missense variants located in the EHMT1 ANKR domain and their initial impact annotations were collected from the ClinVar database.[1] The missense variants were scored with six different sequence-based 2D missense variant impact prediction tools: SNPs&Go,[2] PolyPhen2,[3] PROVEAN,[4] CADD,[5] REVEL,[6] and Rhapsody.[7]

Initial EHMT1 ANKR domain model preparation for in silico analysis

X-ray structure coordinates for the human EHMT1 ANKR domain (amino acid residues 734-967) bound to a 15-mer H3K9me2 peptide (H3 amino acid residues 1-15) (PDB ID 3B95)[8] were obtained from the Protein Data Bank, and the peptide was truncated to 7 amino acid residues (H3 amino acid residues 7-13) as only these residues were ordered in the original X-ray structure. The apo structure of the EHMT1 ANKR domain (PDB ID 6BY9) was used to extend the EHMT1 ANKR domain model to encompass EHMT1 amino acid residues 731-999. Variants were built by substitutions into the energy-minimized apo EHMT1 ANKR domain model (amino acid residues 731-999), and side chain refinement was performed on mutated residues with the ChiRotor algorithm[9] in Discovery Studio suite version 21.1 (Dassault Systèmes BIOVIA). The input models of the apo wild-type and variant ANKR domains were subjected to a two-stage minimization process with Discovery Studio as follows: the energy of the structures was first minimized for 200 steps using the Steepest Descent algorithm, followed by 200 steps of minimization using the Conjugate Gradient algorithm.

In silico protein stability calculations

The impacts of variants on EHMT1 ANKR domain stability were evaluated by calculating the change in folding free energy ($\Delta\Delta G_{folding}$) using two force-field-based methods. The CHARMM36 all-atom forcefield[10] was used to perform alanine scanning mutagenesis ($\Delta\Delta G_{fold\text{-}ALAmut}$), and the impact of variants on ANKR domain stability ($\Delta\Delta G_{fold\text{-}CHARMM}$) was assessed with the Calculate Mutation Energy (Stability) protocol[11] in Discovery Studio. The change in ANKR domain stability for each variant was also evaluated with FoldX ($\Delta\Delta G_{fold\text{-}FOLDX}$).[12,13] The effect of a mutation on protein stability ($\Delta\Delta G_{folding}$) was calculated as the difference between folding free energies ($\Delta G_{folding}$) of the mutant structures ($\Delta G_{folding\text{-}mutant}$) and the WT structures ($\Delta G_{folding\text{-}WT}$).

Structural perturbation assessment

Global and local structure perturbations were assessed by measuring the positional displacement of backbone atoms between the entire ANKR domains of wild-type and variant EHMT1 (global) and only the atoms near the affected residue of interest (local). For global structure perturbation, the entire backbone atoms were used to calculate the RMSD between the wild-type and variant structures. For local structure perturbation, residues within a 10 Å radius from the mutation site were selected with PyMOL version 3.1.3 (Molecular Graphics System, Schrödinger, LLC), and backbone atoms were used for calculating the least-squares RMSD between the wild-type and variant structures with Coot.[14]

Frustration index calculation

To evaluate the energy distribution in the wild-type EHMT1 ANKR domain and how variants shift that energy distribution, the differences in energetic frustration between the wild-type and variant EHMT1 ANKR domains were measured with the Protein Frustratometer server.[15] Frustration index shifts were calculated by summing the positive and negative differences in the frustration indexes between wild-type and missense variant residues. Frustration index shifts were assessed at both global (cumulative) and local levels.

Molecular dynamics (MD) simulations

MD simulations were performed using the CHARMM36 all-atom force field[1074] in Discovery Studio with a 2 fs time step. A simplified distance-dependent implicit solvent environment was used with a dielectric constant of 80 and a pH of 7.4, with no further parameterization of the non-standard residue (K9me2). Models were minimized for 5,000 steps using the Steepest Descent algorithm, followed by an additional 5,000 steps of minimization with the Conjugate Gradient algorithm to relax the protein structure obtained under the stressed crystal environment. Each system of 10 replicates of the wild-type EHMT1 ANKR domain and each variant was independently heated to 300 K over 200 ps and equilibrated for 500 ps, followed by a 10 ns production simulation under the constant number of atoms, volume, and temperature (NVT) ensemble by changing the initial seed (100 ns total). Structures were recorded every 10 ps during the unconstrained dynamics simulation, resulting in a total of 1000 frames for analysis. Frames 501-1000, which reached the near-minimum total energy state, were used for the final data analysis. Select outliers from each data set of 10 replicates that clearly deviate from the rest in RMSD plots, and might represent minor and rarer conformations, were excluded from the final data analysis (Figure S6); at least 7 MD simulation replicates were analyzed per variant, appropriately ensuring reproducibility. Trajectory files from the 10-ns MD simulation

were analyzed for structural impact using RMSD, RMSF, time-dependent molecular interactions, R_g, and SASA. Trajectories were aligned to the initial wild-type conformation before analysis. RMSD and RMSF values were calculated at the residue level for all atoms using the tools available in Discovery Studio and algorithms implemented in Microsoft Excel. Further analyses were carried out in the R programming language[16] using the bio3d package. Molecular visualizations were generated using PyMOL version 3.1.3. MD simulation data were uploaded to the open-access MD data repository MDRepo[17] MDR00021551-MDR00021607).

Time-dependent interaction energy calculations

Using the MD simulation trajectories, followed by the selection of the EHMT1 ANKR domain and interaction groups of interest, the free energies of molecular interactions were measured using the protocols implemented in Discovery Studio. Non-bonded interactions were monitored, and dynamic interaction energies (van der Waals and electrostatic energies) were calculated using the CHARMM36 all-atom force field[10] and the implicit distance-dependent dielectric solvent model.

Surface properties and geometric parameters of the EHMT1 ANKR domain

The dynamic structures from the final frames of individual replicates near the lowest-energy conformation of the trajectories were averaged and used as input for calculations of EHMT1 ANKR domain surface properties and geometric parameters. Surface electrostatic energies and potentials were calculated from MD trajectories using the online Poisson-Boltzmann solver Delphi,[18] with the default Amber force field at pH 7.0, and compared with Coulombic energy values. Solenoid protein-specific geometric terms (*e.g.*, radius of curvature, twist angles) were calculated as previously described using R scripts and the protocols implemented in PyMOL version 3.1.3.[19] End-to-end distances were calculated by selecting the middle point of the first and the last repeating unit. Spring constants were calculated as previously described.[20]

Re-annotation of Kleefstra syndrome-associated EHMT1 ANKR domain missense variants

For overall KS-associated EHMT1 ANKR domain missense variant impact re-annotation relative to existing ClinVar annotations, individual scores from each level of the integrated (2D/3D/4D) structural genomics analysis were converted to Z-scores, then scaled from 0 to 1, combined with equal weights, and averaged to obtain the IMFS (Table S1).[21] EHMT1 ANKR domain missense variants were re-annotated based on IMFS (IMFS<0.2: tolerated, 0.2<IMFS<0.28: variant of uncertain significance, and IMFS>0.28: damaging). Variant annotation thresholds

were determined by ROC based on IMFS and H3K9me1 peptide binding (variant binding with H3K9me1 FC $K_d \geq 0.5$) (Figure S4, Table S4) to maximize sensitivity and specificity according to Youden's index.[22] ROC analyses were conducted in GraphPad Prism, and 95% confidence intervals were determined using the Wilson/Brown method.

EHMT1 ANKR domain vector construct and mutagenesis

The His_6-tagged human EHMT1 ANKR domain construct (amino acids 672-999) in a pET28-MHL expression vector was a gift from Cheryl Arrowsmith (Addgene plasmid #119956). Variants were generated by site-directed mutagenesis (GenScript) and confirmed by Sanger sequencing.[23]

Protein expression and purification

The recombinant wild-type and variant EHMT1 ANKR domains were purified from BL21(DE3) *E. coli* by nickel-affinity chromatography. BL21(DE3) cells were transformed and grown at 37 °C in Terrific Broth media with 50 mg/L kanamycin to an absorbance of 0.6-1.0 at 600 nm. Protein expression was induced overnight with 0.1-1 mM IPTG at 18 °C. Cells were harvested by centrifugation at 5000 × *g*, and cell pellets were frozen at -80 °C until lysis.

Frozen cells were thawed on ice and resuspended in lysis buffer (50 mM K_3PO_4, 500 mM NaCl, 10 mM imidazole, 5% v/v glycerol, pH 7.4) supplemented with 0.1 mg/mL lysozyme, 5 µg/mL DNase I, 5 mM $MgCl_2$, 14 µM β-mercaptoethanol, and 1× Halt Protease Inhibitor Cocktail (Thermo Fisher Scientific) or a cocktail of 0.3 nM aprotinin, 1 nM E-64, 1 nM leupeptin, 1 nM bestatin, 1 nM pepstatin A, and PMSF protease inhibitors. Resuspended cells were immediately lysed by sonication for 10 minutes (pulsed, amplitude 3.5, 50% work cycle). Lysates were clarified by centrifugation for 30 minutes at 30,000 rpm, and 4 °C. Clarified lysates were then applied to nickel-nitrilotriacetic acid (Ni-NTA) resin (~1 mL beads/L culture) or Ni-charged MagBeads (GenScript, L00295) (~1 mL beads/L culture) at 4 °C for at least 1 hour while rocking.

Protein-bound Ni-NTA resins were applied to a column, washed with 3 × 15 mL lysis buffer, and then eluted with 5 mL fractions of elution buffer (50 mM K_3PO_4, 500 mM NaCl, 5% v/v glycerol, pH 7.4) with 50, 100, 150, 200, and 250 mM imidazole. Fractions were resolved by SDS-PAGE, and those containing the EHMT1 ANKR domain were pooled. Protein-bound Ni-charged MagBeads were washed with 3 × 10 mL lysis buffer containing 0.02% v/v Tween 20, followed

by 3 × 10 mL lysis buffer alone. Proteins were eluted from the beads with 5 mL of elution buffer with 250 mM imidazole using the AmMag SA Plus semi-automated purification system (GenScript, L01013).

Protein samples were further purified by gel filtration size-exclusion chromatography using an XK 16/40 column (Cytiva, 28988938) or a HiLoad 16/600 Superdex 75 column (Cytiva, 28989333) into storage buffer (50 mM K_3PO_4, 500 mM NaCl, 5% v/v glycerol, pH 7.4). For all protein purifications, monomeric proteins were collected based on the chromatograms resulting from gel filtration size-exclusion chromatography. Concentrations of purified proteins were determined by measuring the absorbance at 280 nm and using the extinction coefficient of the wild-type EHMT1 ANKR domain construct (32,930 M^{-1} cm^{-1}) determined with ProtParam.[24] The proteins were then aliquoted, flash-frozen, and stored at -80 °C.

Nano differential scanning fluorimetry (nanoDSF)

Wild-type and variant EHMT1 ANKR domains were dialyzed into nanoDSF buffer (50 mM K_3PO_4, 500 mM NaCl, pH 7.4), gently stirring overnight at 4 °C using 3.5 kDa molecular weight cutoff Slide-a-lyzer mini dialysis units (Thermo Fisher Scientific, 69550). Protein samples were prepared at a concentration of 50-100 μM in nanoDSF buffer. Samples were drawn up in triplicate in Prometheus NT.48 high-sensitivity capillaries (NanoTemper Technologies, PR-C006) and run at 10-50% excitation power with a temperature ramp of 20 to 95 °C, increasing 0.5 °C/minute, in a Prometheus NT.38 instrument (NanoTemper Technologies, PR001). The ratio of intrinsic Trp fluorescence at 350 nm to intrinsic Tyr fluorescence at 330 nm was plotted versus temperature over the course of the temperature ramp. Protein melting temperatures were determined by identifying the maximum of the first derivative of the fluorescence ratio with ThermControl software version 2.3.1.

Circular dichroism

Wild-type and variant EHMT1 ANKR domains were dialyzed into CD buffer (20 mM Na_3PO_4, 50 mM NaF, pH 7.5), gently stirring overnight at 4 °C using 3.5 kDa molecular weight cutoff Slide-a-lyzer mini dialysis units (Thermo Fisher Scientific, 69550). Secondary structure was assessed using a J1500 CD spectrophotometer (JASCO). Protein samples were prepared at 0.2 mg/mL in CD buffer and placed in 1 mm quartz cuvettes (JASCO J/0556). CD spectra were recorded at 25 °C from 190 to 260 nm with a data pitch of 0.1 nm. A bandwidth of 1 nm was used with a digital integration time of 8 seconds and a scanning speed of 10 nm/min. Each spectrum was

accumulated from 5 scans. The data were processed using Spectra Analysis software supplied by the manufacturer (JASCO) and transferred to GraphPad Prism for presentation.

Preparation of fluorescent 5-TAMRA-H3K9me1 and H3K9me2 peptides

15-mer mono- (NH_2-ARTKQTARK(me1)STGGKA-Pra-NH_2) and di-methylated (NH_2-ARTKQTARK(me2)STGGKA-Pra-NH_2) H3K9 peptides with alkyne handles (Pra = propargylglycine) were synthesized by Peptide 2.0. 5-TAMRA azide (AAT Bioquest, 486) was added to the H3K9 peptides using copper-catalyzed azide-alkyne cycloaddition click chemistry as follows. Each peptide (1 mg) was separately dissolved in 400 µL of ddH_2O in black 1.7 mL tubes, then mixed with 5 mM 5-TAMRA azide and 500 mM HEPES pH 7.0 for a final concentration of 0.73 mM 5-TAMRA azide and 25 mM HEPES pH 7.0. Equal volumes of 20 mM $CuSO_4$ and 100 mM 2-(4-((bis((1-(tert-butyl)-1H-1,2,3-triazol-4-yl)methyl)amino)methyl)-1H-1,2,3-triazol-1-yl)acetic acid (BTTAA) were mixed by gentle pipetting in a clear 1.7 mL tube until the solution turned blue. 100 mM sodium ascorbate was added to the $CuSO_4$/BTTAA solution until the solution turned clear. The $CuSO_4$/BTTAA/sodium ascorbate solution was combined with the peptide/5-TAMRA azide/HEPES solution in black 1.7 mL tubes and incubated at room temperature with rotation for at least 15 minutes. The click reaction was quenched by diluting the entire volume to 1,500 µL, yielding a final concentration of 1% v/v acetonitrile and 0.1% v/v trifluoroacetic acid. Fluorescent 5-TAMRA-H3K9 peptides were purified by reversed-phase high-performance liquid chromatography (HPLC) using a C18 column and a 0-40% gradient of acetonitrile in 0.1% v/v trifluoroacetic acid in H_2O. Elution fractions containing the fluorescent peptides were then concentrated using a rotary evaporator, dried to a powder with a V-10 Touch evaporation system (Biotage), and stored at -20 °C protected from light.

Fluorescence polarization (FP)

Wild-type and variant EHMT1 ANKR domains were dialyzed into FP buffer (25 mM Tris, 140 mM NaCl, 3 mM KCl, 0.1% v/v Tween 20, pH 8.0), gently stirring overnight at 4°C using 3.5 kDa molecular weight cutoff Slide-a-lyzer mini dialysis units (Thermo Fisher Scientific, 69550). Protein samples were prepared at a concentration range of 0.08-200 µM in FP buffer using serial dilution. Dried fluorescent 5-TAMRA-H3K9me1 and H3K9me2 peptides were dissolved in sterile ddH_2O to a concentration of approximately 5 mM, then further diluted to 200 nM in FP buffer. Peptide concentrations at each stage were determined by measuring the absorbance at 552 nm and using the extinction coefficient of the 5-TAMRA dye (AAT Bioquest, 486) provided by the manufacturer (90,000 M^{-1} cm^{-1}). Equal volumes of 0.08-200 µM protein samples and 200

nM 5-TAMRA-H3K9me1 or H3K9me2 peptides were mixed in triplicate in 384 black-well plates for a final 12-point titration curve at protein concentrations of 0.04-100 µM protein with 100 nM peptides. Fluorescence polarization values were measured with a BioTek Cytation 5 imaging reader (Agilent, 16277) using a red FP filter cube (Agilent, 8040562) with excitation at 530 nm and emission at 590 nm. FP polarization values were transferred to GraphPad Prism and fit using quadratic non-linear regression (Equation 1) to determine the methylated H3K9 peptide binding affinity (K_d):

$$P_{obs} = P_{free} + \frac{(P_{bound} - P_{free}) \times (([A] + [L]_{tot} + K_d) - \sqrt{(([A] + [L]_{tot} + K_d)^2 - (4 \times [A] \times [L]_{tot}))}}{(2 \times [L]_{tot})} \quad \text{(Eq. 1)}$$

where P_{obs} is the observed fluorescence polarization value, [A] is the protein (ANKR domain) concentration, $[L]_{tot}$ is the total fluorescent ligand (5-TAMRA-H3K9me1 or H3K9me2 peptide) concentration, P_{free} is the FP polarization value of the free peptide, and P_{bound} is the FP polarization value of the peptide fully bound to protein. K_d values for variants are reported in terms of FC relative to the K_d values of the wild-type EHMT1 ANKR domain binding to the H3K9me1 and H3K9me2 peptides, respectively.

Sequence Alignment

Sequences of the ANKR domains of EHMT1 and EHMT2 were obtained from UniProt[25] in FASTA format and submitted to the Clustal Omega multiple sequence alignment program.[26] The resulting alignment was analyzed and edited using ESPript 3.0[27] and Adobe Illustrator. Canonically conserved ankyrin repeat residues were identified from prior studies.[28]

Supplemental Text

Text S1. R script used to evaluate solenoid geometry.

```
#1. Install and load required packages
library(bio3d)

#2. Define the Euclidean distance function, which calculates the straight-
line distance between two points in 3D space
euclidean <- function(a, b) sqrt(sum((a - b)^2))

#3. Define the function that calculates the radius of curvature from
residues; inputs are the path to the PDB file and the three residues used
for this calculation
radCurv <- function(pdbFile = NULL, resTriplet = NULL){

#4. Read the PDB structure into Bio3D
p <- read.pdb(pdbFile)

#5. Select the 3 residues used to calculate the geometry
  #coord of side 1
  S1<- resTrip[1]
  #coord of center
  C <- resTrip[2]
  #coord of side 2
  S2 <- resTrip[3]

#6. Extract the Cα atoms
  caAtom <- p$atom[p$calpha,]

#7. Find the first side residue
  S1Logical <- caAtom$resno == S1

#8. Find the center residue
  CLogical <- caAtom$resno == C

#9. Find the second side residue
  S2Logical <- caAtom$resno == S2

#10. Extract the three specific Cα atoms
  CaS1 <- caAtom[S1Logical,]
  CaC <- caAtom[CLogical,]
  CaS2 <- caAtom[S2Logical,]

#11. Extract the 3D coordinates defining the geometry used in the
curvature calculation
  coordCaS1 <- c(CaS1$x, CaS1$y, CaS1$z)
  coordCaC <- c(CaC$x, CaC$y, CaC$z)
  coordCaS2 <- c(CaS2$x, CaS2$y, CaS2$z)

#12. Calculate the Cα-Cα distance between S1 and C (d1)
  d1 <- euclidean(coordCaC, coordCaS1 )

#13. Calculate the Cα-Cα distance between C to S2 (d2)
```

```
  d2 <- euclidean(coordCaC, coordCaS2)

#14. Calculate the average of the S1-C and the C-S2 distances
  d <- mean(c(d1, d2))

#15. Calculate half of the distance between S1 and S2 (m)
  m <- (euclidean(coordCaS1, coordCaS2))/2

#16. Calculate the radius of curvature (R) and round the result to 4
decimal places
  R <- (d^2)/(2 * sqrt(d^2 - m^2))
  return(round(R, 4))

}
#======================Do Not edit above this line========================#
# Declaring the variables

#A. Specify the location of the PDB files
pdbp <- file.path('path/to/folder/containing/target/file')

#B. Find the PDB files
pdbl <- list.files(pdbp, pattern = "_991.pdb")

#C. Define the residue triplet
#side center side
resTrip <- c(115, 228, 374)
#These three numbers can be substituted with residue numbers of interest

#D. Create an empty results table and calculate the radius of curvature
from every PDB file in pdbl, and input the results into the table
df <- data.frame(PdbFile=character(0),
                 RadiusCurv = numeric(0))

for(i in seq_along(pdbl)){
  pdbf <- file.path(pdbp, pdbl[i])
  RadCurv <- radCurv(pdbFile = pdbf, resTriplet = resTrip)
  df[i, 1] <- pdbl[i]
  df[i, 2] <- RadCurv
}

#E. Print the final results table
print (df)

##End of analysis
```

Text S2. R script used to generate the Figure 2 correlation matrices.

```
#Instructions: The target file should be in Excel format.

#1. Change working directory to the folder containing your target file
setwd("path/to/folder/containing/target/file")

#2. Install and load required packages
library(readxl)
library(dplyr)

#3. Import the Excel file into R and display the entire dataset
df<-read_excel("path/to/folder/containing/target/file")
df

#4. Select the variables for the correlation analysis and display the
selected variables
df1<-select(df, SNPsGo, PROVEAN, PolyPhen2, REVEL, CADD, Rhapsody, FoldX,
StabilityEnergy, GlobalPerturb, LocalPerturb, GlobalFrustration,
CoulombicEnergy, TwistAngle, RadCurv, K9Me2Interaction,
PeptideInteraction, GluK9Me2Distance, RMSD.median.diff, RMSD.avg.diff,
RMSD.avg.abs.diff, Inv.RMSF.Scorr, Inv.RMSF.Pcorr, RMSF.avg.diff,
RMSF.avg.abs.diff, RMSF.abs.res.sum, Rg, SASA)
df1

#df1<-select(df, PolyPhen2, REVEL, CADD, Rhapsody, FoldX, StabilityEnergy,
GlobalPerturb, LocalPerturb, GlobalFrustration, CoulombicEnergy,
TwistAngle, RadCurv, PeptideInteraction, Rg)
##Another selection used; can be substituted for the selection above

#5. Convert blank cells to NA and display the cleaned data
df1[df1==""]  <- NA
df1
#where NA indicates a missing value

#6. Remove rows containing missing values and display the cleaned data
df2<-select(na.omit(df1), SNPsGo, PROVEAN, PolyPhen2, REVEL, CADD,
Rhapsody, FoldX, StabilityEnergy, GlobalPerturb, LocalPerturb,
GlobalFrustration, CoulombicEnergy, TwistAngle, RadCurv, K9Me2Interaction,
PeptideInteraction, GluK9Me2Distance, RMSD.median.diff, RMSD.avg.diff,
RMSD.avg.abs.diff, Inv.RMSF.Scorr, Inv.RMSF.Pcorr, RMSF.avg.diff,
RMSF.avg.abs.diff, RMSF.abs.res.sum, Rg, SASA)
df2

#df2<-select(na.omit(df1), PolyPhen2, REVEL, CADD, Rhapsody, FoldX,
StabilityEnergy, GlobalPerturb, LocalPerturb, GlobalFrustration,
CoulombicEnergy, TwistAngle, RadCurv, PeptideInteraction, Rg)
##Another selection used; can be substituted for the selection above

#7. Load the corrplot package
library(corrplot)
```

```
#8. Calculate the Spearman correlation matrix
spearman_corr <- cor(df2, method = "spearman")

#9. Display the final correlation matrix
corrplot(spearman_corr, type = "upper",
        tl.col = "dodgerblue3", tl.cex = 0.9, font =1.8, number.font = 2,
method = "square", addCoef.col = "black",
        number.cex = 0.50,
        diag = FALSE)

##End of analysis
```

Text S3. R script used to generate the Figure 4 PCA plot.

```
#Instructions: The target file should be in Excel format. Rows in the
target file correspond to protein variants, and columns correspond to
numeric variables. The first column should contain variant names (e.g.,
WT, missense variants).

#1. Change working directory to the folder containing your target file
setwd("path/to/folder/containing/target/file")

#2. Install and load required packages
install.packages(c("readxl", "tidyverse", "factoextra"))

library(readxl)
library(tidyverse)
library(factoextra)

#3. Load Excel data
data <- read_excel("variant_data.xlsx", sheet = 1)

data_df <- data %>%
column_to_rownames(var = colnames(data)[1])

data_numeric <- data_df %>% select(where(is.numeric))
#where "variant_data.xlsx" is the name of your target file

#4. Run PCA (scaled)
pca_res <- prcomp(data_numeric,
center = TRUE,
scale. = TRUE)

#5. Generate PCA plot of variants
fviz_pca_ind(
pca_res,
repel = TRUE,
geom = "point",
pointsize = 3,
col.ind = "steelblue"
)

#6. Generate PCA biplot (variants + variables)
fviz_pca_biplot(
pca_res,
repel = TRUE,
col.var = "firebrick",
col.ind = "steelblue",
arrowsize = 1
)

#7. Determine variable contributions to principal components
var_contrib <- get_pca_var(pca_res)$contrib
print(var_contrib)
fviz_contrib(pca_res,
choice = "var",
```

```
axes = 1,
top = 10)

fviz_contrib(pca_res,
choice = "var",
axes = 2,
top = 10)

#8. Export variable contributions
write.csv(var_contrib,
"PCA_variable_contributions.csv")

##End of analysis
```

Supplemental Tables

Table S1. Spreadsheet of the 12 selected individual prediction methods and group layer scores, scaled Z-scores, and molecular fitness scores.

Molecular fitness scores are determined by combining and averaging the scaled Z-scores from each metric with equal weights. Sub-total scores are independently calculated for 2D, 3D, 4D, and 3D/4D scores. Variants purified and tested biophysically are bolded in the Table. HGVS nomenclature is included for all variants analyzed in the study.

Table S2. EHMT1 ANKR domain wild-type and missense variant T_m and ΔT_m values.

EHMT1 ANKR domain	T_m (°C)	ΔT_m (°C)
WT	41.4 ± 0.01	0
P709L	42.0 ± 0.07	0.6 ± 0.07
K733R	42.4 ± 0.02	1.0 ± 0.02
K753E	41.7 ± 0.01	0.3 ± 0.01
A780T	35.0 ± 0.05	-6.5 ± 0.05
A783S	39.9 ± 0.03	-1.6 ± 0.02
V793L	41.7 ± 0.05	0.3 ± 0.06
P809L	ND	ND
A837S	42.3 ± 0.1	0.9 ± 0.1
G839V	ND	ND
T881A	37.6 ± 0.02	-3.8 ± 0.02
T881I	42.3 ± 0.7	0.9 ± 0.7
G896D	35.0 ± 0.03	-6.4 ± 0.04
E923K	37.3 ± 0.1	-4.1 ± 0.1
A927T	36.9 ± 0.2	-4.5 ± 0.2
L976R	37.6 ± 0.2	-3.8 ± 0.2
N982S	38.7 ± 0.2	-2.8 ± 0.2
S983Y	34.9 ± 0.03	-6.5 ± 0.03

Values were determined by nanoDSF, where error represents standard deviation from samples run in technical triplicate. ND = not determined.

Table S3. Binding affinity (K_d) of EHMT1 ANKR domain wild-type and missense variants to H3K9me1 and H3K9me2 peptides.

EHMT1 ANKR domain	K_d H3K9me1 (μM)	K_d H3K9me2 (μM)
WT	1.4 ± 0.1	2.4 ± 0.1
W879A	*>100	*>100
E882A	*>100	*>100
K753E	3.6 ± 0.2	5.1 ± 0.3
A783S	2.2 ± 0.3	6.9 ± 0.9
V793L	1.5 ± 0.1	3.8 ± 0.2
P809L	*>100	*>100
A837S	1.5 ± 0.2	4.2 ± 0.3
G839V	29.6 ± 7.2	30.5 ± 7.1
T881A	2.7 ± 0.5	8.3 ± 0.9
T881I	*>100	*>100
G896D	*>100	*>100
E923K	*>100	*>100
A927T	*>100	*>100
S983Y	13.6 ± 1.9	45.8 ± 18.9
A780T	2.4 ± 0.4	17.4 ± 4.1
L976R	0.4 ± 0.02	1.4 ± 0.1
N982S	0.3 ± 0.02	1.0 ± 0.1

Values were determined in FP experiments; error represents the standard deviation from fitting samples run in technical triplicate. For the wild-type EHMT1 ANKR domain, error represents the standard deviation from fitting samples run in technical triplicate across two experimental replicates. * = error not determined due to no binding observed with up to 100 μM protein.

Table S4. Sensitivity and specificity from ROC with IMFS and H3K9me1 peptide binding.

Threshold	Sensitivity	Sensitivity 95% CI	Specificity	Specificity 95% CI	Youden Index (J)
0.15	1	0.68-1	0.14	0.01-0.51	0.14
0.17	1	0.68-1	0.29	0.05-0.64	0.29
0.18	1	0.68-1	0.43	0.16-0.75	0.43
**0.20**	1	0.68-1	0.57	0.25-0.84	**0.57**
0.21	0.88	0.53-0.99	0.57	0.25-0.84	0.45
**0.28**	0.88	0.53-0.99	0.71	0.36-0.95	**0.59**
0.44	0.75	0.41-0.96	0.71	0.36-0.95	0.46
0.54	0.63	0.31-0.86	0.71	0.36-0.95	0.34
0.55	0.63	0.31-0.86	0.86	0.49-0.99	0.48
0.57	0.50	0.22-0.78	0.86	0.49-0.99	0.36
0.60	0.50	0.22-0.78	1	0.65-1	0.50
0.64	0.38	0.14-0.69	1	0.65-1	0.38
0.67	0.25	0.04-0.59	1	0.65-1	0.25
0.74	0.13	0.01-0.47	1	0.65-1	0.13

The optimal thresholds and Youden indices are bolded.

Table S5. Percent contributions of integrated structural genomics analysis metrics to the first two components of EHMT1 ANKR domain missense variant PCA.

Analysis Metric	PC1 (%)	PC2 (%)
PROVEAN	6.8	3.4
PolyPhen	7.3	0.0
REVEL	7.2	0.2
Rhapsody	7.1	0.5
2D score	7.8	0.5
FoldX	5.8	9.6
StabilityEnergy	5.3	10.9
LocalPerturb	5.2	1.0
GlobalFrustration	4.7	0.2
3D score	7.1	3.1
Coulombic Energy	0.7	29.9
TwistAngle	3.7	19.1
PeptideInteraction	5.2	3.7
R_g	4.0	0.0
4D score	5.8	17.0
3D/4D score	8.0	1.0
IMFS	8.4	0.0

Supplemental Figures

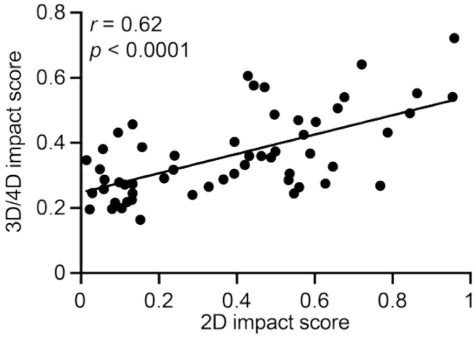

Figure S1. Correlation between Kleefstra syndrome-associated missense variant 2D impact scores and 3D/4D impact scores.

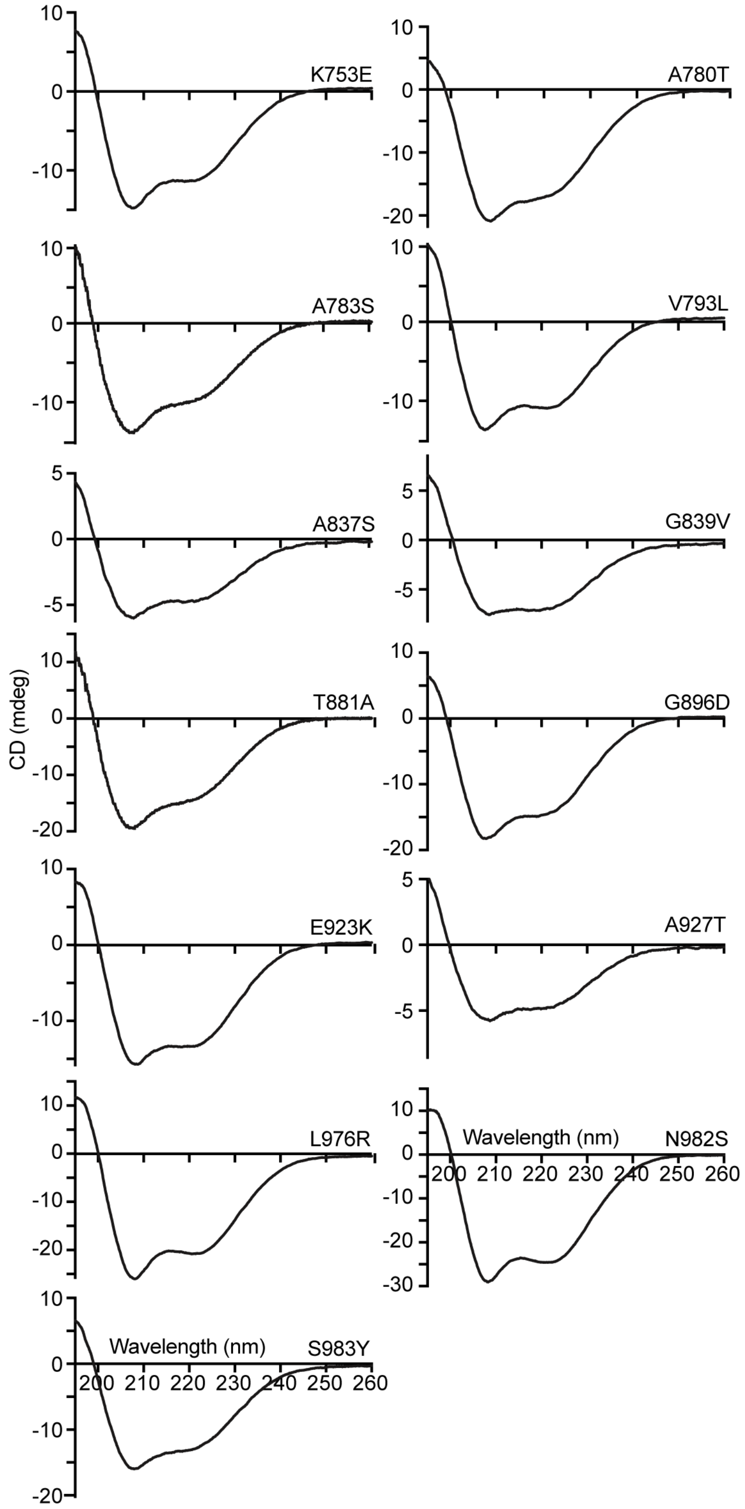

Figure S2. Circular dichroism spectra of Kleefstra syndrome-associated EHMT1 ANKR domain missense variants.

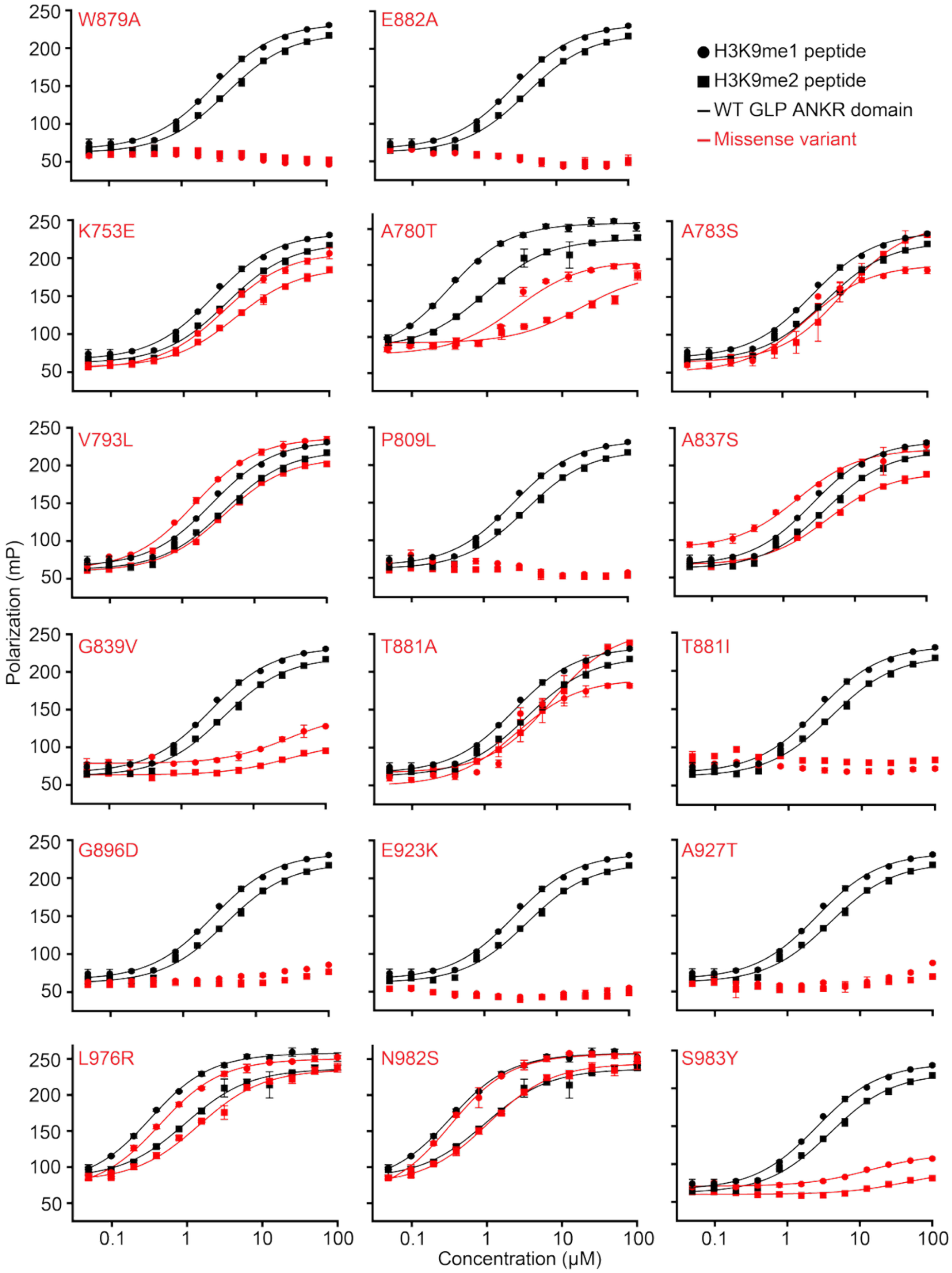

Figure S3. FP titrations of the wild-type and missense variant EHMT1 ANKR domains (0.04 – 100 µM) binding to fluorescent 5-TAMRA-labeled H3K9me1 or H3K9me2 peptides (100 nM).

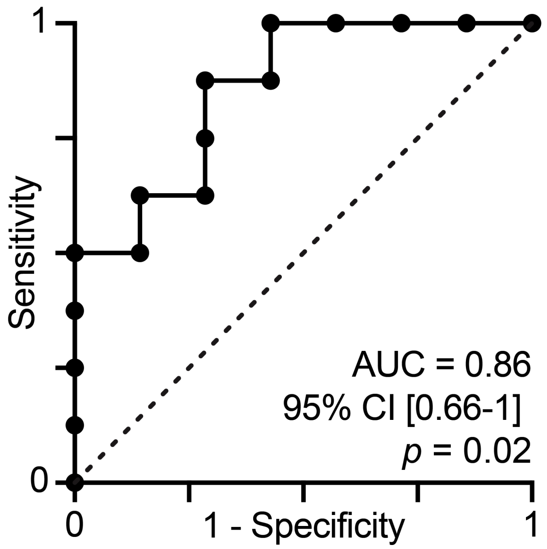

Figure S4. Receiver operating characteristic (ROC) analysis for score threshold determination. ROC analysis of all soluble EHMT1 ANKR domain variants based on IMFS and H3K9me1 peptide binding (variant binding with H3K9me1 FC $K_d \geq 0.5$) to determine the thresholds for tolerated vs. VUS vs. damaging variant annotation.

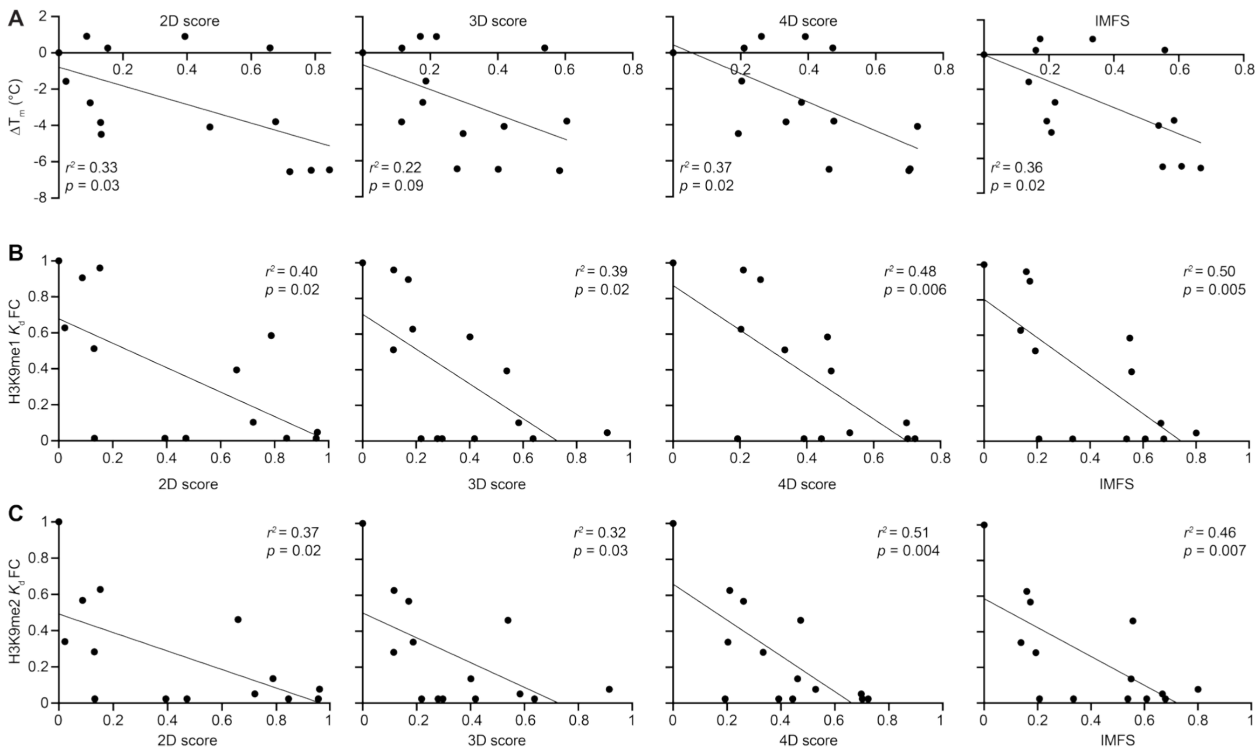

Figure S5. Correlation between structural genomics analysis level scores and EHMT1 ANKR domain missense variant ligand stability and ligand binding as assessed by nanoDSF (ΔT_m) and FC (fold change of the K_d for H3K9me1 and H3K9me2 peptides compared to the wild-type EHMT1 ANKR domain, where K_d FC <2).

(A) Score correlations with ΔT_m. (B) Score correlations with H3K9me1 K_d FC. (C) Score correlations with H3K9me2 K_d FC.

As scores from each layer of the structural genomics analysis were determined in reference to the wild-type EHMT1 ANKR domain, wild-type EHMT1 is included in the plots with a ΔT_m of 0 °C and a K_d FC of 1.

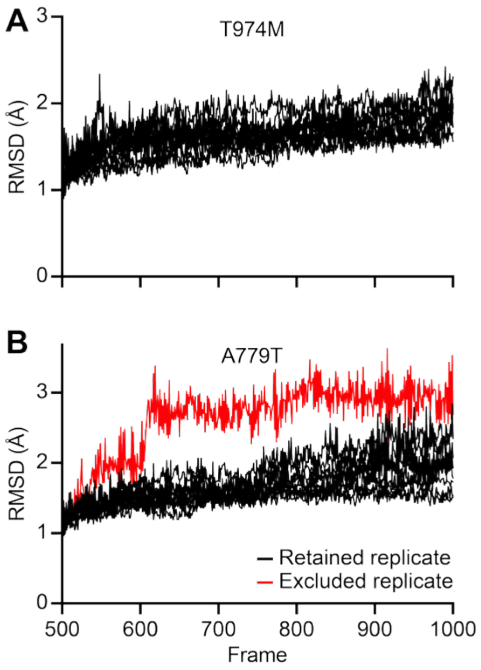

Figure S6. Representative RMSD graphs for replicate inclusion vs. exclusion.
(A) EHMT1 T974M, a representative variant where all replicates were included in the analysis based on RMSD. (B) EHMT1 A779T, a representative variant where select replicates were excluded from the analysis based on RMSD.

Supplemental References

1. Landrum, M.J. et al. ClinVar: public archive of relationships among sequence variation and human phenotype. *Nucleic Acids Res* **42**, D980-5 (2014).
2. Calabrese, R., Capriotti, E., Fariselli, P., Martelli, P.L. & Casadio, R. Functional annotations improve the predictive score of human disease-related mutations in proteins. *Hum Mutat* **30**, 1237-44 (2009).
3. Adzhubei, I.A. et al. A method and server for predicting damaging missense mutations. *Nature Methods* **7**, 248-249 (2010).
4. Choi, Y. & Chan, A.P. PROVEAN web server: a tool to predict the functional effect of amino acid substitutions and indels. *Bioinformatics* **31**, 2745-2747 (2015).
5. Rentzsch, P., Witten, D., Cooper, G.M., Shendure, J. & Kircher, M. CADD: predicting the deleteriousness of variants throughout the human genome. *Nucleic Acids Research* **47**, D886-D894 (2018).
6. Ioannidis, N.M. et al. REVEL: An Ensemble Method for Predicting the Pathogenicity of Rare Missense Variants. *The American Journal of Human Genetics* **99**, 877-885 (2016).
7. Ponzoni, L., Peñaherrera, D.A., Oltvai, Z.N. & Bahar, I. Rhapsody: predicting the pathogenicity of human missense variants. *Bioinformatics* **36**, 3084-3092 (2020).
8. Collins, R.E. et al. The ankyrin repeats of G9a and GLP histone methyltransferases are mono- and dimethyllysine binding modules. *Nat Struct Mol Biol* **15**, 245-50 (2008).
9. Spassov, V.Z., Yan, L. & Flook, P.K. The dominant role of side-chain backbone interactions in structural realization of amino acid code. ChiRotor: a side-chain prediction algorithm based on side-chain backbone interactions. *Protein Sci* **16**, 494-506 (2007).
10. Best, R.B. et al. Optimization of the Additive CHARMM All-Atom Protein Force Field Targeting Improved Sampling of the Backbone ϕ, ψ and Side-Chain $\chi1$ and $\chi2$ Dihedral Angles. *Journal of Chemical Theory and Computation* **8**, 3257-3273 (2012).
11. Spassov, V.Z. & Yan, L. A pH-dependent computational approach to the effect of mutations on protein stability. *Journal of Computational Chemistry* **37**, 2573-2587 (2016).
12. Guerois, R., Nielsen, J.E. & Serrano, L. Predicting Changes in the Stability of Proteins and Protein Complexes: A Study of More Than 1000 Mutations. *Journal of Molecular Biology* **320**, 369-387 (2002).
13. Shihab, H.A. et al. Predicting the functional, molecular, and phenotypic consequences of amino acid substitutions using hidden Markov models. *Hum Mutat* **34**, 57-65 (2013).
14. Emsley, P. & Cowtan, K. Coot: model-building tools for molecular graphics. *Acta Crystallogr D Biol Crystallogr* **60**, 2126-32 (2004).
15. Parra, R.G. et al. Protein Frustratometer 2: a tool to localize energetic frustration in protein molecules, now with electrostatics. *Nucleic Acids Res* **44**, W356-60 (2016).
16. Team RC. R: A language for environmental and statistical computing. *R Foundation for Statistical Computing, Vienna, Austria* **https://www.R-project.org/.2020.(2020**).
17. Roy, A. et al. MDRepo-an open data warehouse for community-contributed molecular dynamics simulations of proteins. *Nucleic Acids Res* **53**, D477-d486 (2025).
18. Sarkar, S. et al. DelPhi Web Server: A comprehensive online suite for electrostatic calculations of biological macromolecules and their complexes. *Commun Comput Phys* **13**, 269-284 (2013).
19. Geraldo, M.T., Takeda, A.A., Braz, A.S. & Lemke, N. Bending-Twisting Motions and Main Interactions in Nucleoplasmin Nuclear Import. *PLoS One* **11**, e0157162 (2016).
20. Kappel, C., Zachariae, U., Dölker, N. & Grubmüller, H. An unusual hydrophobic core confers extreme flexibility to HEAT repeat proteins. *Biophys J* **99**, 1596-603 (2010).
21. Chi, Y.I. et al. Structural bioinformatics enhances the interpretation of somatic mutations in KDM6A found in human cancers. *Comput Struct Biotechnol J* **20**, 2200-2211 (2022).

22. Schisterman, E.F., Faraggi, D., Reiser, B. & Hu, J. Youden Index and the optimal threshold for markers with mass at zero. *Stat Med* **27**, 297-315 (2008).
23. Sanger, F., Nicklen, S. & Coulson, A.R. DNA sequencing with chain-terminating inhibitors. *Proc Natl Acad Sci U S A* **74**, 5463-7 (1977).
24. Gasteiger, E. et al. Protein Identification and Analysis Tools on the ExPASy Server. in *The Proteomics Protocols Handbook* (ed. Walker, J.M.) 571-607 (Humana Press, Totowa, NJ, 2005).
25. UniProt: the Universal Protein Knowledgebase in 2023. *Nucleic Acids Res* **51**, D523-d531 (2023).
26. Sievers, F. et al. Fast, scalable generation of high-quality protein multiple sequence alignments using Clustal Omega. *Mol Syst Biol* **7**, 539 (2011).
27. Robert, X. & Gouet, P. Deciphering key features in protein structures with the new ENDscript server. *Nucleic Acids Res* **42**, W320-4 (2014).
28. Izert, M.A., Szybowska, P.E., Górna, M.W. & Merski, M. The Effect of Mutations in the TPR and Ankyrin Families of Alpha Solenoid Repeat Proteins. *Front Bioinform* **1**, 696368 (2021).
